# Probing the Influence of the Protein Scaffold on H-Cluster Reactivity via Gain-of-Function Studies—Improved H_2_ Evolution and O_2_ Tolerance through Rational Design of [FeFe] Hydrogenase

**DOI:** 10.1021/jacs.4c17364

**Published:** 2025-01-27

**Authors:** Princess R. Cabotaje, Alina Sekretareva, Moritz Senger, Ping Huang, Kaija Walter, Holly J. Redman, Nicholas Croy, Sven T. Stripp, Henrik Land, Gustav Berggren

**Affiliations:** † Molecular Biomimetics, Department of Chemistry, Ångström Laboratory, Uppsala University, P.O. Box 523, Uppsala SE-75120, Sweden; ‡ Biochemistry, Department of Chemistry, Biomedical Centre, Uppsala University, Uppsala SE-75120, Sweden; § Spectroscopy and Biocatalysis, Institute of Chemistry, Universität Potsdam, Potsdam D-14476, Germany

## Abstract

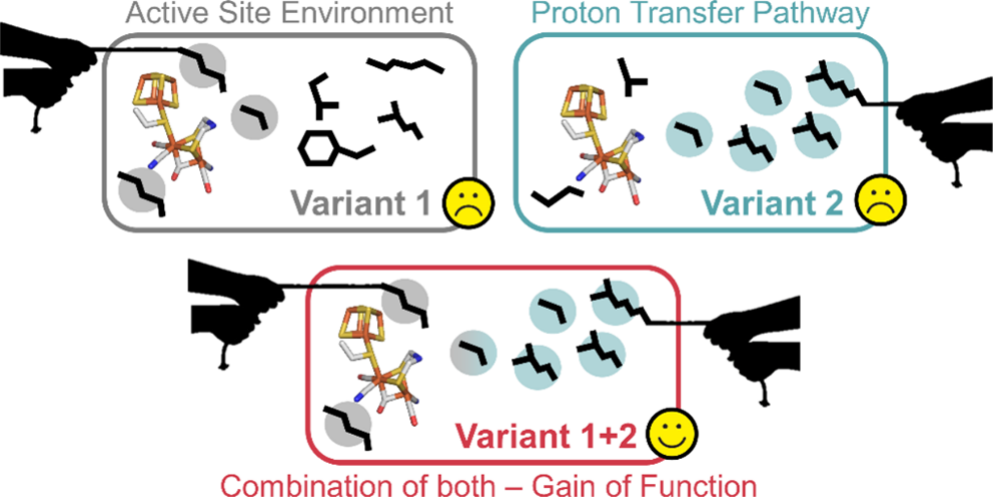

[FeFe] hydrogenases make up a structurally diverse family of metalloenzymes that catalyze proton/dihydrogen interconversion. They can be classified into phylogenetically distinct groups denoted A–G, which differ in structure and reactivity. Prototypical Group A hydrogenases have high turnover rates and remarkable energy efficiency. As compared to Group A enzymes, the putatively sensory Group D hydrogenase from *Thermoanaerobacter mathranii* (*Tam*HydS) has a thousand-fold lower H_2_ evolution rate and a high overpotential requirement to drive catalysis (irreversible) but shows increased inhibitor tolerance. This divergence in structure and activity between hydrogenases makes them ideal models for studying second (active-site environment) and outer (*e.g.*, substrate transport) coordination sphere effects on metal cofactors. Herein, we generated three *Tam*HydS-based variants, each mimicking proposed key structural features of Group A hydrogenase: the “active site” (**AS**), “proton-transfer pathway” (**PTP**), and “combined” (**CM** = **AS** + **PTP**) variant. A fourth single-point variant, **A137C**, which introduces a proposed critical cysteine in the active site, was characterized as a reference. No change in isolation resulted in Group A-like behavior; *i.e*., no positive impact on catalytic performance was observed. The **CM** variant, however, showed increased H_2_ evolution activity but retained the overpotential requirement. Additionally, the **CM** variant improved the already relatively high stability of *Tam*HydS against O_2_ and CO inhibition. These findings show that activity rates, (ir)reversibility, and susceptibility to gaseous inhibitors are decoupled. Moreover, the results highlight the importance of exploring hydrogenase diversity as a path toward understanding the structural factors that enable the outstanding catalytic properties of [FeFe] hydrogenases.

## Introduction

Hydrogenases are metalloenzymes serving central functions in energy metabolism, where they catalyze the interconversion of protons (H^+^), electrons, and hydrogen gas (H_2_).^1,2^ The so-called [FeFe] hydrogenases are generally considered as the most active, with turnover frequencies (TOFs) of 10^4^–10^5^ s^–1^ reported for H_2_ evolution and oxidation.^3,4^ Remarkably, the enzyme can sustain these rates very close to the thermodynamic potential of the reaction. Consequently, [FeFe] hydrogenases have attracted considerable attention in the context of renewable energy research and serve as an inspiration for the design of synthetic H_2_-evolving catalysts.^5,6^

**Figure 1:**
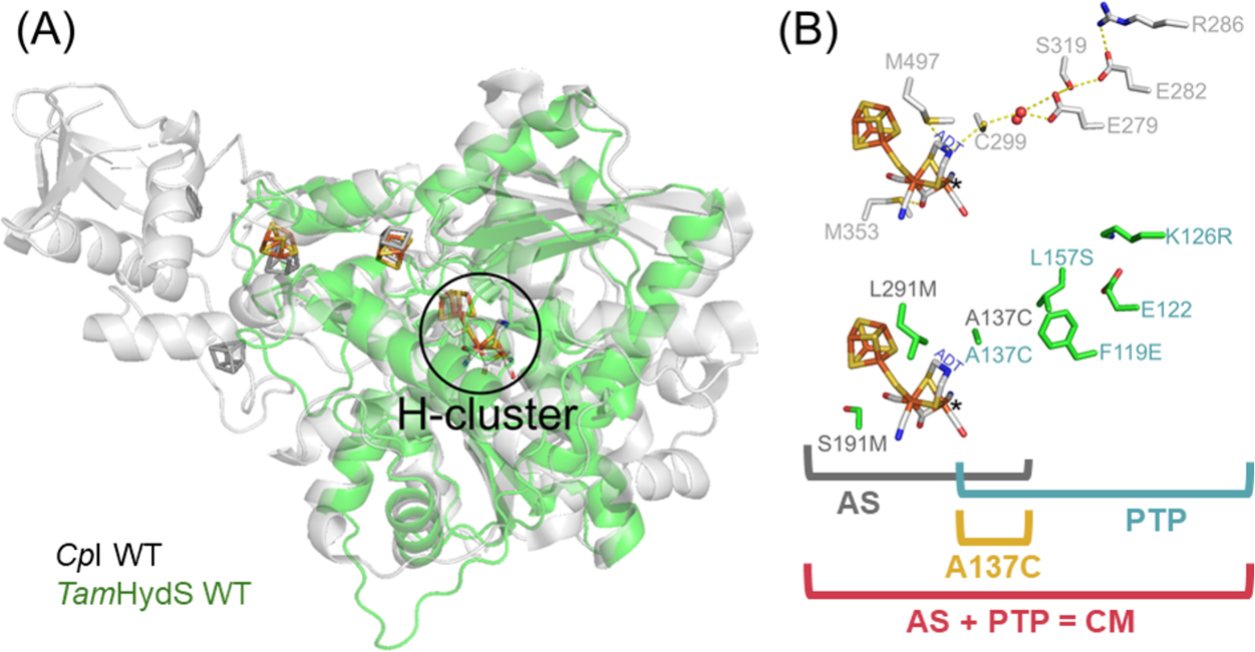
Structural comparison between *Tam*HydS and the prototypical [FeFe] hydrogenase *Cp*I. (A) Alignment of the YASARA-generated homology model (Alignment RMSD: 1.541 Å) of *Tam*HydS^17^ (green cartoon) with the crystal structure of *Cp*I (gray cartoon, PDB ID: 4XDC(18)), including an overlay of F-clusters (orange: Fe, yellow: S for *Tam*HydS, gray for *Cp*I). The H-cluster is encircled in solid black. Note: one [4Fe-4S]-cluster is not modeled here. Figure S1 presents the AlphaFold model of *Tam*HydS, including the third accessory [4Fe-4S]-cluster located in the C-terminal domain. (B) Top: Amino acids (gray: C, red: O, blue: N) constituting the proton-transfer pathway (C299, E279, S319, E282, and R286) or interacting with the H-cluster through H-bonding and other electrostatic interactions (C299, M353, and M497) in *Cp*I. Hydrogen turnover is catalyzed at Fe_d_ (*), *i.e*., the iron ion most distal to the [4Fe-4S]_H_ cluster. The azadithiolate (ADT) bridgehead is labeled as blue text. Bottom: The corresponding residues from *Tam*HydS (green: C, red: O, blue: N). The following variants were investigated: (1) single-point variant **A137C**, (2) “active site” or **AS** (A137C, L291M, and S191M in gray labels); (3) “proton-transfer pathway” or **PTP** (A137C, F119E, L157S, and K126R in cyan labels); and (4) “combination” of **AS** and **PTP** variations or **CM**. The glutamate residue (E122) in *Tam*HydS was not substituted, given that the analogous residue in *Cp*I, E282, is conserved as glutamate across both enzymes.

A unifying feature of all [FeFe] hydrogenases is their organometallic active-site cofactor, the “H-cluster” (Figure 1A). This biologically unique cofactor consists of a canonical iron–sulfur cluster ([4Fe-4S]_H_) linked via a bridging cysteinyl thiolate to a diiron subsite ([2Fe]_H_) with CO and CN^–^ ligands that stabilize the iron ions in a low-spin state.^7−11^ The two iron ions of the [2Fe]_H_ subsite are further ligated by a bridging azadithiolate ligand (^−^SCH_2_NHCH_2_S^–^, ADT).^12,13^ [FeFe] hydrogenases often feature additional ferredoxin-type iron–sulfur clusters, commonly referred to as “F-clusters”. These clusters serve as molecular wires enabling electron transfer between the H-cluster and external redox partners, and tune the catalytic properties of the enzyme.^14−16^

In addition to the inorganic cofactors, the influence of the protein matrix on [FeFe] hydrogenase catalysis and inhibition has been extensively studied.^19^ Structural and functional studies, in combination with site-directed mutagenesis, have revealed how the second coordination sphere controls the spatial arrangement of the ligands (*i.e*., CO, CN^–^, ADT) and tune the reactivity of the H-cluster.^20−23^ Moving beyond the active site, the protein also ensures controlled delivery of protons and gaseous reactants, which further impacts the enzyme’s overall catalytic performance.^24,25^ Still, the structural factors that enable the remarkable reactivity of [FeFe] hydrogenase remain opaque. The role of second and outer coordination sphere effects is also receiving attention in the design of synthetic systems for H_2_ catalysis.^26−28^ Thus, improved understanding of the biological blueprints is expected to also benefit molecular design efforts.

To date, studies of the second coordination sphere have focused primarily on the structurally similar “prototypical” [FeFe] hydrogenases of Group A, including *Chlamydomonas reinhardtii* HydA1 (*Cr*HydA1), *Clostridium pasteurianum* HydA1 (*Cp*I), and the closely related *Clostridium acetobutylicum* HydA1 (*Ca*HydA1), as well as the dimeric enzyme from *Desulfovibrio desulfuricans* (*Dd*HydAB).^29^ However, [FeFe] hydrogenases form a structurally diverse enzyme family, which can be divided into distinct phylogenetic groups denoted as Group A–G.^2,29−32^ The immediate protein environment around the H-cluster is conserved in each group, but can vary significantly between groups.^29,33^ Similarly, the proton-transfer pathway is altered between groups.^17,34−36^ Comparative studies between different groups of [FeFe] hydrogenase are expected to provide a wealth of new information concerning the role of the protein scaffold in tuning the hydrogenase activity.

The putatively sensory [FeFe] hydrogenase from *Thermoanaerobacter mathranii* (*Tam*HydS) is an example of a Group D enzyme.^17^ In comparison to the previously studied Group A [FeFe] hydrogenases, *Tam*HydS displays significantly lower H_2_ evolution rates. Moreover, although *Tam*HydS functions as a bidirectional catalyst under electrochemical conditions, both proton reduction and H_2_ oxidation display significant overpotentials.^17,37^ Such irreversible bidirectional behavior is rare among reported hydrogenases and related molecular catalysts.^29,37−40^*Tam*HydS also displays unusual reactivity toward known hydrogenase inhibitors. Previously studied Group A [FeFe] hydrogenases show a complete loss of the H-cluster upon contact with O_2_,^41−43^ with few group members as exceptions.^42,44,45^ Meanwhile, exposing *Tam*HydS to O_2_ transforms the [2Fe]_H_ subsite into a mononuclear species, which is stable under prolonged exposure to O_2_ and retains limited H_2_ evolution capacity.^17^ In addition to its unusual reactivity toward O_2_, *Tam*HydS was found to have a significantly higher tolerance toward CO. An elevated CO tolerance has also been reported for *Tm*HydS from *Thermotoga maritima*, a representative example of a Group C [FeFe] hydrogenase (Table S1).^34^ As the H-cluster is identical in all groups, the reactivity differences must be attributed to variations in second and/or outer coordination sphere, *e.g*., the active-site environment surrounding the H-cluster and/or the proton-transfer pathway.^17,35^

Considering second coordination sphere effects, methionine residues M497_*Cp*I_ and M353_*Cp*I_ (*Cp*I numbering; see Figure 1B), are proposed to be critical in modulating the reactivity of the H-cluster in Group A [FeFe] hydrogenases.^21,38^ In the case of *Tam*HydS, these residues are exchanged to leucine (L291_*Tam*HydS_) and serine (S191_*Tam*HydS_), respectively. Additionally, cysteine residue C299_*Cp*I_, which serves as the entry point into the well-conserved proton-transfer pathway of Group A,^25^ is not conserved in Group D [FeFe] hydrogenases and is exchanged to alanine in *Tam*HydS (A137_*Tam*HydS_). The corresponding cysteine-to-alanine variants in Group A enzymes (C169A_CrHydA1,_^46,47^ C299A_CpI_,^24^ and C298A_*Ca*HydA1_^48^) exhibit reduction in H_2_ turnover, highlighting the cysteine residue’s importance in catalysis.^49^ Except for a glutamic acid (E122_*Tam*HydS_ and E282_*Cp*I_), all residues considered crucial for proton transfer in *Cp*I (C299, E279, S319, and R286)^25,50,51^ are exchanged in *Tam*HydS (to A137, F119, L157, and K126, respectively). An alternative proton-transfer pathway has been proposed that is conserved within Group D, but variable in other groups.^35^ This striking difference in the proton-transfer pathway, or outer coordination sphere, was previously proposed as a possible factor rationalizing the irreversible catalytic behavior of *Tam*HydS.^37^ However, kinetic modeling has revealed that the irreversibility can be assigned to a one-electron reduced H-cluster intermediate, H_red_, being stable over an unusually large potential range.^52^ This latter proposal is further supported by spectro-electrochemical titrations performed on the structurally related Group C enzyme, *Tm*HydS.^34^

The diverging structural features and catalytic properties of Group A and Group D [FeFe] hydrogenases provide a new entry point for investigating the complex interplay between the H-cluster and protein environment. Herein, we utilize the design principles of Group A [FeFe] hydrogenase to improve the catalytic performance of the Group D [FeFe] hydrogenase, *Tam*HydS, via rational design. Three new enzyme constructs based on *Tam*HydS were generated to mimic key features strictly conserved in Group A [FeFe] hydrogenases. As summarized in Figure 1B, these constitute an “active-site” (**AS**) variant and a “proton-transfer pathway” (**PTP**) variant. The two variants recreate important features of the active-site pocket and proton-transfer pathway of a prototypical [FeFe] hydrogenase, respectively. The third variant combines these modifications and is denoted the “combined” (**CM**) variant. For reference, a variant introducing only the active-site cysteine (A137C_*Tam*HydS_, equivalent to C299_*Cp*I_) was characterized (**A137C**). Critically, this approach allowed us to investigate the design principles of hydrogenase via gain-of-function variations rather than loss-of-function studies. Through a combination of spectroscopic, electrochemical, and biochemical analyses, we show that mimicking neither the Group A active site nor the proton-transfer pathway in isolation has a positive impact on the catalytic performance of *Tam*HydS. Only the combined modification generates a hybrid enzyme displaying aspects of both Group A and Group D hydrogenases. Compared to *Tam*HydS wild-type (**WT**), the **CM** variant improved H_2_ evolution rates up to 170-fold, accompanied by improved stability toward inhibition by CO and O_2_.

## Results

### Isolation and Spectroscopic Characterization of Variants

**A137C**, **AS**, **PTP**, and **CM** variants were generated based on a comparison between *Tam*HydS and the crystal structure of *Cp*I (Figure 1).^18^ The **AS** triple variant introduces two methionine residues (S191M and L291M) and the proton relay cysteine (A137C). The **PTP** quadruple variant includes the proton relay cysteine (A137C) in combination with additional proton-transfer pathway variations (F119E, K126R, and L157S). The **CM** sextuple variant incorporates all of the aforementioned variations. Lastly, the reference variant, **A137C**, featured only the proton relay cysteine mutation. The individual genetic constructs were transformed into BL21(DE3) *Escherichia coli* cells to produce the respective *apo*-protein, *i.e*., a form of the hydrogenase lacking the [2Fe]_H_ subsite (Figures S2 and S3). As no changes were made in the F-cluster binding domains, the variants are expected to incorporate three [4Fe-4S] clusters in addition to the [4Fe-4S]_H_ cluster. Albeit the fact that cells were lysed and the enzyme was isolated under anaerobic conditions, the iron content of the as-isolated variants differed slightly. Still, all variants shared the same final Fe:protein ratio of 15.9 ± 0.2 after reconstitution (Table S2).^17^ The successful assembly of *Tam*HydS’ four [4Fe-4S] clusters was further confirmed by UV/visible spectroscopy and, in the case of **AS**, **PTP**, and **CM**, also by electron paramagnetic resonance (EPR) spectroscopy (Figures S4 and S5). The EPR spectra recorded on dithionite-reduced *apo*-forms of all constructs revealed two rhombic EPR signals attributable to the F-clusters ([4Fe-4S]^+^, *S* = 1/2) highly similar to that previously reported for **WT**,^17^ showing that the variations did not significantly alter the electron transfer relays (Figure S5). The reconstituted *apo*-proteins were subsequently treated with the synthetic [2Fe]_H_ site precursor [(ADT)Fe_2_(CN)_2_(CO)_4_]^2–^ [([2Fe]^ADT^)] and purified using desalting columns to generate the corresponding *holo*-enzymes. The Fe:protein ratio of the **WT** and variants was 17.9 ± 0.4, in good agreement with the addition of two iron ions in the active-site cofactor (Table S2).

**Figure 2:**
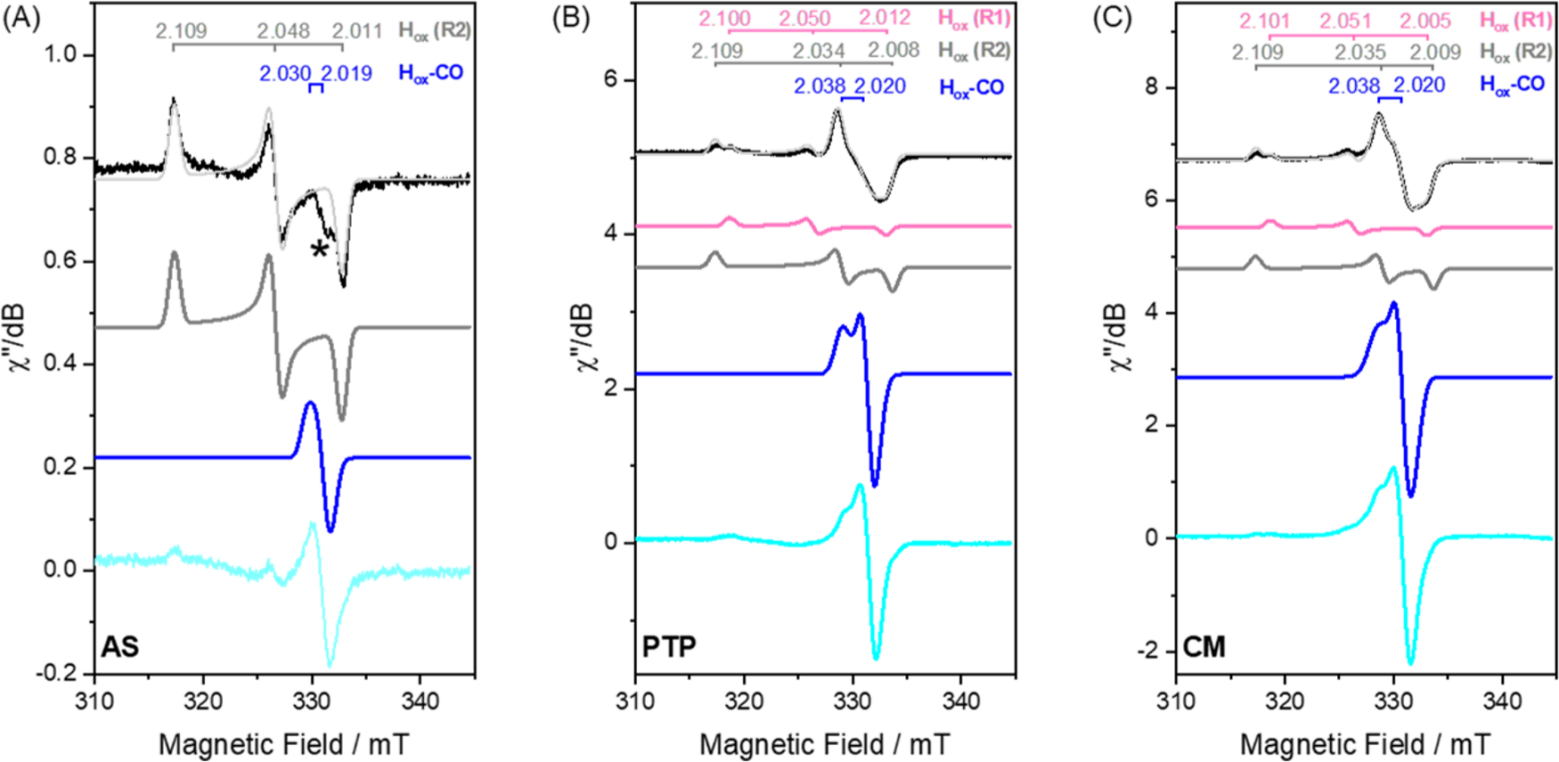
EPR characterization of *Tam*HydS variants. EPR spectra of the as-isolated (black) and CO-flushed (cyan) *Tam*HydS variants (A) **AS**, (B) **PTP**, and (C) **CM**. The simulations for the as-isolated spectra are depicted as light gray traces overlaid with the respective experimental spectrum. All spectra reveal features attributable to the H-cluster residing in the H_ox_ state (component H_ox_-R1 *g*-values are shown in magenta; component H_ox_-R2 *g*-values are shown in dark gray). The axial signal is attributed to H_ox_-CO (*g*-values in blue), which increases in the signal amplitude after an hour of flushing with CO. Spectra recorded with the following settings: *T* = 21 K; modulation frequency = 100 kHz; amplitude = 10 G; microwave frequency = 9.4 GHz. Microwave power: 16 μW for all spectra, with the exception of the spectrum of the CO-flushed **PTP** recorded at 253 μW. The individual components contributing to the overall simulated spectra are shown in magenta (H_ox_-R1, simulated from black spectra), dark gray (H_ox_-R2, simulated from black spectra), and blue (H_ox_-CO), simulated from cyan spectra with the exception of **PTP** for which the H_ox_-CO simulation was derived from the spectrum of CO-flushed **PTP** recorded at 64 μW (Figure S6D). The asterisk (*) in panel A corresponds to a trace signal from a [3Fe-4S]^+^-cluster (*g* ≈ 2.02; see Figure S6).

**Figure 3:**
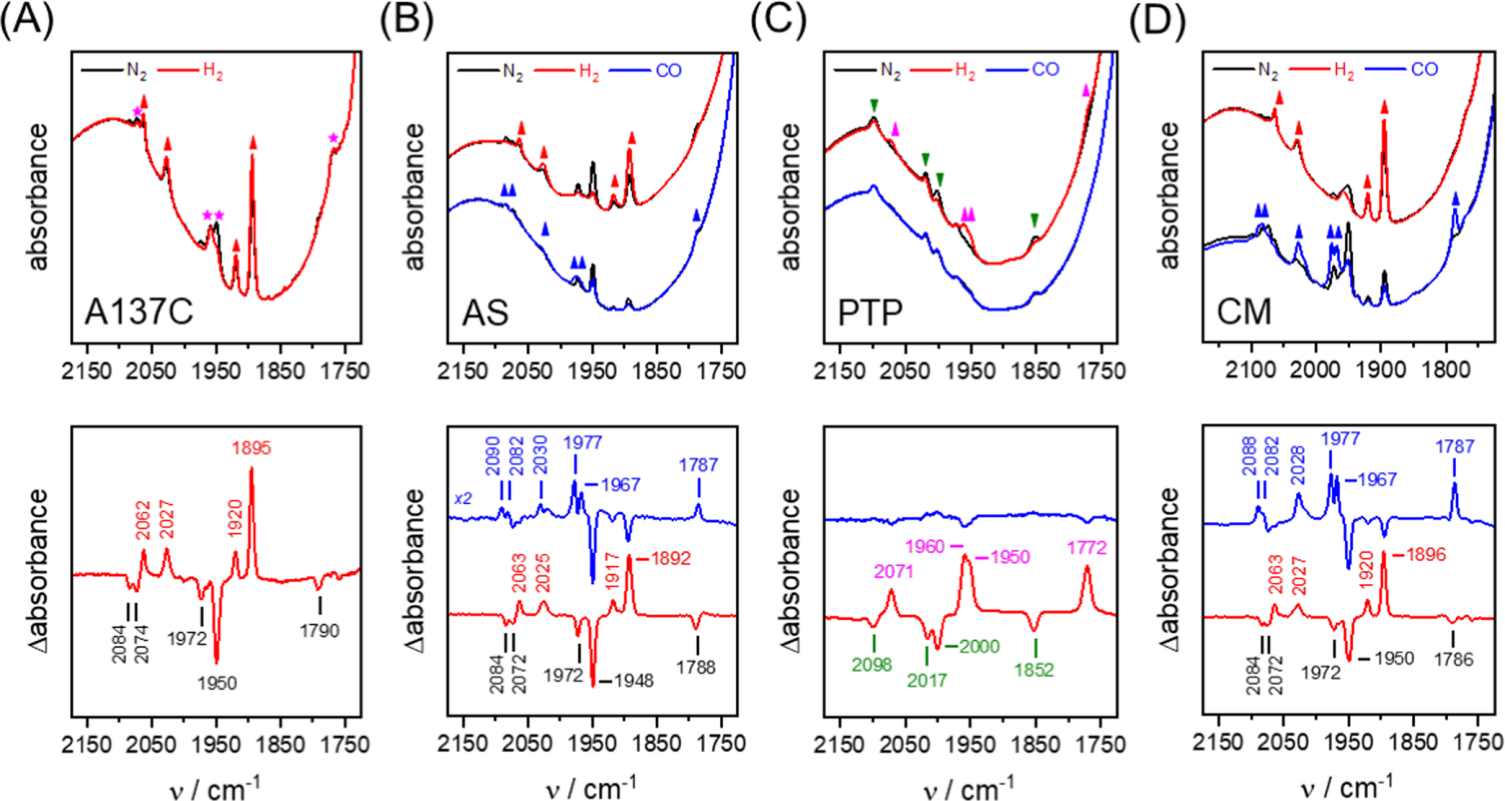
ATR-FTIR characterization of *Tam*HydS variants. Infrared spectra of (A) **A137C**, (B) **AS**, (C) **PTP**, and (D) **CM** for the reaction with H_2_ (red traces) and CO (blue traces). The upper row shows absolute spectra, and the lower row shows the resulting “H_2_–N_2_” and “CO–N_2_” difference spectra. The H-cluster states observed under N_2_ (black labels, H_ox_), H_2_ (red labels, H_red_), and CO (blue labels, H_ox_-CO) are annotated. **A137C**, **AS**, and **CM** adopt H_red_ during H_2_ exposure. **AS** and **CM** adopt H_ox_-CO when exposed to CO. Arrows pointing upward (↑) or downward (↓) highlight increasing or decreasing bands. The magenta stars in the absolute spectrum of **A137C** indicate the band positions for **State 2**. The transition from **State 1** to **State 2** observed in the **PTP** “H_2_–N_2_” difference spectrum is annotated in green and magenta, respectively. Note that **PTP** in **State 1** does not bind to CO. A summary of the bands associated with the H_ox_, H_ox_-CO, and H_red_ states together with a comparison to Group A enzymes is provided in Table S4. All data were recorded at room temperature.

EPR and *in situ* attenuated total reflection Fourier transformed infrared (ATR-FTIR) spectroscopy supports the formation of the H-cluster in all variants (Figures 2 and 3). In agreement with earlier studies,^17^ the reference EPR spectrum of a freshly activated sample (denoted “as-isolated”) of **WT** displayed a mixture of H-cluster states in the *g* ∼ 2 region (Table S3). Similar preparations of **PTP** and **CM** showed two highly similar rhombic “resting state” H_ox_ components (Figure 2B,C, black spectra). Simulation of the spectra revealed the following *g*-values for **PTP** H_ox_-R1: *g*_1,2,3_ = 2.100, 2.050, 2.012; H_ox_-R2: *g*_1,2,3_ = 2.109, 2.034, 2.008; for **CM** H_ox_-R1: *g*_1,2,3_ = 2.101, 2.051, 2.005; for **CM** H_ox_-R2: *g*_1,2,3_ = 2.109, 2.035, 2.009 (simulated spectra of H_ox_-R1 and H_ox_-R2 are shown as magenta and dark gray traces in Figure 2, respectively). There was also a significant contribution by an axial signal attributed to the CO-inhibited state H_ox_-CO (*g*_II_ = 2.038, *g*_⊥_ = 2.020, simulated H_ox_-CO spectra are shown as blue traces in Figure 2). As expected, the latter signal dominated the spectra recorded of samples incubated under a CO atmosphere (Figure 2B,C, cyan spectra). The H_ox_-CO signals from **PTP** and **CM** displayed a highly similar set of *g*-values but differed in their relaxation properties (Figure S6A,B). As-isolated **AS** presented the simplest spectrum, with the dominant signal similar to the H_ox_-R2 state of **WT**, with *g*_1,2,3_ = 2.109, 2.048, and 2.011 (Figure 2A, black spectrum). An axial signal attributed to H_ox_-CO was observable for **AS** after prolonged exposure to CO (*g*_II_ = 2.030, *g*_⊥_ = 2.019; Figure 2A, cyan spectrum). The narrower, more isotropic H_ox_-CO signal in **AS**, compared to those in **PTP** and **CM**, partially overlaps with the residual [3Fe-4S]^+^ signal. However, its distinct shape and relaxation characteristics allow for differentiation (Figure S6C). Overall, the observed *g*-values for the H_ox_ and H_ox_-CO states exhibit a high degree of similarity across the variants, with the most notable alterations observed in **AS**. The *g*-values consistently remain higher than those reported for prototypical [FeFe] hydrogenases (Table S3).

In all three variants, incubating the enzyme under 1 atm of H_2_ resulted in the expected decrease in H_ox_ and H_ox_-CO signal intensities. Instead, new signals appeared, consisting of broad rhombic features attributable to reduced [4Fe-4S]-cluster species in combination with a narrow axial signal (Figure S7). The latter signal is assigned to a state denoted as “**State 2**”. **State 2** refers to a still only partially characterized H-cluster state that forms in **WT***Tam*HydS, as well as previously studied variants, under H_2_. Earlier studies have proposed the **State 2** signal to reflect a species with a redox-state configuration similar to H_ox_ (*i.e*., an oxidized [4Fe-4S]_H_-cluster coupled to a mixed valent Fe(I)Fe(II) [2Fe]_H_ site; see Supporting Note 1 for further discussion on this state).^35^ Albeit present in all samples, differences are observed between the variants of *Tam*HydS with regard to the intensity of the **State 2** signal, implying the presence of contrasting amounts of reduced EPR silent species such as H_red_ (Figure S7). Similar to the H_ox_ signals, comparing the simulated EPR spectra of H_2_-reduced **CM** and **WT** revealed that the **State 2** signals are highly similar, indicating only minor shifts in their electronic structure.

The IR signatures of typical H-cluster states (H_ox_, H_red_, and H_ox_-CO) were verified in the absolute and difference spectra of **AS** and **CM** and again differ only slightly from **WT***Tam*HydS (Figures 3, S8 and Table S4), supporting the EPR data. As previously observed for **WT**,^17^ both **AS** and **CM** adopted H_red_ as a semistable resting state when exposed to the H_2_-containing atmosphere of the glovebox housing the FTIR instrument (1% H_2_ in 99% N_2_). Purging under a neat N_2_ atmosphere resulted in the conversion of H_red_ into the oxidized species, H_ox_. The rate of H-cluster oxidation differed between the variants but consistently occurred on a time-scale of hours (Figure S9). Exposure to H_2_ reformed the H_red_ state on a seconds time-scale, although **AS** and **CM** reacted with H_2_ 2–8 times slower than **WT** (Figure S10). Similarly, formation of H_ox_-CO was found to be 3–5 times slower than **WT** when the enzyme films were exposed to a neat CO atmosphere (99% CO, 1% N_2_, Figures S9 and S10). No H_ox_-CO formation was observed when 1% H_2_ was included in the headspace gas (data not shown).

Conversely, for **PTP**, only a negligible population of the H-cluster resided in the H_ox_ and H_red_ states. Instead, the spectrum was dominated by a signature that is strikingly similar to a species denoted **State 1**, recently observed in other variants of *Tam*HydS targeting the enzyme’s native proton-transfer pathway (Figure 3C).^35^ The exact nature of **State 1** remains to be elucidated, but it is likely to represent an “over-oxidized” form of the H-cluster similar, but not identical, to H_inact_ (*i.e*., an oxidized [4Fe-4S]_H_-cluster coupled to a diferrous [2Fe]_H_ site). Formation of **State 1** in the **PTP** variant can be promoted through incubation under both a CO containing as well as a neat N_2_ atmosphere, and consequently does not appear to involve binding of extraneous CO. As **State 1** is also readily formed in the absence of an external sulfide, and in *Tam*HydS variants lacking any cysteines in the active site, binding of a thiol ligand to the [2Fe]_H_ site can be ruled out. Instead, we have previously proposed that the [2Fe]_H_ subsite is coordinated by an aqua or hydroxido ligand (see also Supporting Note 1).^35^ Reduction of **PTP** by H_2_ yielded a spectrum typical of **State 2**.

While there is overall a good correlation between the FTIR and EPR data in the case of **AS** and **CM**, noticeable differences are observed for the **PTP** variant. **State 1** dominates the FTIR spectrum, while the EPR spectrum of as-isolated **PTP** (Figure 2B) shows the distinct signatures of H_ox_ and H_ox_-CO. This is at least partially attributable to the EPR silent (*S* = 0) nature of **State 1**, preventing its detection by EPR. By extension, this in turn facilitates detection of the mixed valent H_ox_ and H_ox_-CO states by EPR even if present in relatively low concentrations. The apparent absence of these states in FTIR is instead attributed to their rapid loss upon exposure of the enzyme to the H_2_-containing atmosphere of the glovebox housing the FTIR instrument. However, we note that changes in state populations arising from differences in sample conditions cannot be ruled out, e.g., the large temperature differences in EPR and FTIR (cryogenic vs room temperature, respectively).

Lastly, the **A137C** variant displayed a mixture of three H-cluster states (Figure 3A). Analogous to the **CM** and **AS** variants, the FTIR spectrum was dominated by signals that could be attributed to reduced species. More specifically, **A137C** featured a significant population of H_red_, but in contrast to **CM** and **AS**, a large population of **State 2** was also evident in the spectrum. A minor H_ox_ population was also discernible. The band signatures of the H_red_ and H_ox_ signatures were similar to those observed in **WT**, **AS**, and **CM**, while the **State 2** bands align with those observed in **PTP** (Figure 3) and our earlier study.^35^ Exposure of the **A137C** variant to higher H_2_ concentrations yielded an increase in the H_red_ population with concurrent depopulation of H_ox_, while the **State 2** signal remained practically unchanged.

A summary of the observed FTIR bands associated with the H_ox_, H_ox_-CO, and H_red_ states together with selected reference values reported for Group A [FeFe] hydrogenases is shown in Table S4. Overall, recreating either the active-site second coordination sphere (**AS**) or the proton-transfer pathway (**PTP**) resulted in variants with clearly distinct properties. **AS** gave rise to a single form of H_ox_, in contrast to the two forms observed for **WT**. On the other hand, **PTP** yielded an enzyme that appeared to be dominated by H-cluster species **State 1** and **State 2**, while **A137C** featured a mixture of **State 2** and H_red_. Clearly, altering the second and outer coordination sphere impacts the relative stability of different H-cluster states in *Tam*HydS, in line with earlier work on Group A [FeFe] hydrogenases.^24,53,54^ In *Tam*HydS, a tendency to preferentially populate **State 1** and **State 2** appears to be primarily associated with changes in outer coordination sphere residues connected to proton transfer, as observed here with the **PTP** variant and to a lesser extent in **A137C**, as well as in earlier work.^35^ However, **CM** restored the spectroscopic properties of **WT**, although the kinetics governing the formation of various states were altered.

### Catalytic Properties of Variants

The catalytic properties of the variants were evaluated using a combination of solution assays and protein film electrochemistry (PFE) to probe the effect of the variations on specific activity, apparent catalytic bias, and overpotential (η) for H_2_ oxidation and proton reduction. **WT** was included in all of the assays as a reference.

Cyclic voltammetry (CV) traces were recorded following enzyme adsorption on a pyrolytic graphite electrode (PGE) in the presence of polycationic polymyxin B sulfate, as previously reported.^17^ To ensure that the observed currents closely approximate steady-state conditions, the potential was swept at a slow scan rate (2 mV/s). Substrate mass transport limitations for H_2_ oxidation at high overpotentials were alleviated by saturating the cell with constant H_2_ flow (1 atm) and by rotating the electrode at high speed (3000 rpm).

**Figure 4:**
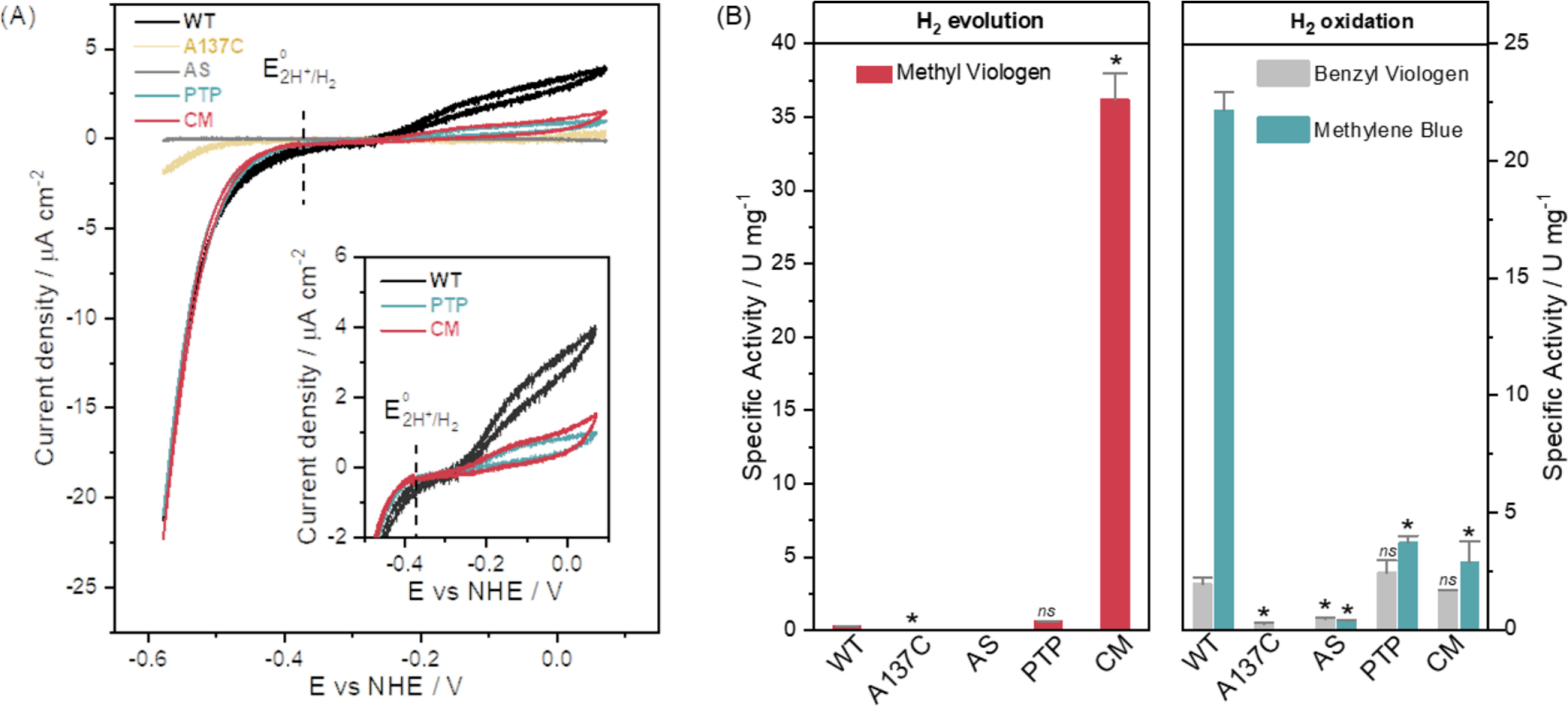
Catalytic properties of *Tam*HydS WT enzyme and variants. (A) Cyclic voltammetry (CV) traces (first scan) recorded for **WT** (black), **A137C** (gold), **AS** (light gray), **PTP** (cyan), and **CM** (red) subtracted with the blank electrode (no immobilized enzyme). Inset: Zoom in near the formal reduction potential of the H^+^/H_2_ couple, emphasizing the apparent oxidative inactivation of **PTP** and **CM** at higher applied potentials. Second scans and current densities as a function of the scan number are shown in Figures S11 and S12. Experimental conditions: pH 6.0, 40 °C, 1 atm H_2_, scan rate 2 mV/s, electrode rotation 3000 rpm. (B) Comparison of the specific activities for H_2_ oxidation and evolution at pH 6.8 using methyl viologen (MV: 10 mM MV + 100 mM sodium dithionite, red bars), benzyl viologen (BV: 1 mM, gray bars), and methylene blue (MB: 30 μM, cyan bars). H_2_ oxidation was measured in colorimetric assays at 25 °C and H_2_ evolution by gas chromatography at 28 °C. Specific activity (U/mg) is one unit (U) of activity that catalyzes 1 μmol of H_2_ oxidized or evolved per minute per mg of enzyme. One-way ANOVA (Tukey’s Honestly Significant Difference Test) results are indicated above the bars: significantly different at the *p* = 0.05 level (*) and not significantly different (ns) based on mean exam scores between **WT** and variants (Table S5). Data are presented as a mean of two biological replicates, each with 2–3 technical replicates with error bars showing the standard error of the mean (SEM). Not detected: H_2_ evolution rate in **AS**. Not determined was H_2_ oxidation of **A137C** using MB.

The CV traces, recorded under conditions of direct electron transfer (DET), clearly show that both **CM** and **PTP** are bidirectional catalysts, displaying catalytic currents related to both proton reduction and H_2_ oxidation (Figure 4A). Under the conditions of the PFE experiment, both variants displayed a significant increase in the apparent catalytic bias relative to **WT**, *i.e*., the preference to catalyze the 2H^+^/H_2_ interconversion in a specific direction. Indeed, the ratio of the proton reduction rates relative to H_2_ oxidation increases 2–3-fold in **CM** and **PTP** when compared to **WT**, as determined from the currents observed at a high driving force with η ≈ 200 mV. More specifically, under the employed conditions, 40 °C and a pH of 6.0, the *E*°′ is approximately −0.37 V vs the normal hydrogen electrode (NHE), and the currents were determined at *E* = −0.57 and −0.15 V vs NHE for proton reduction and H_2_ oxidation, respectively. This increase in the apparent bias toward proton reduction appears at least partially attributable to the inhibition of **CM** and **PTP** at strongly oxidizing potentials. This is observable as a decrease in current despite an increase in the driving force, apparent from approximately *E* > −0.12 V vs NHE (see also Figure S13). It is also in line with the more sluggish reactivity of **CM** toward H_2_ oxidation as observed in the time-resolved FTIR experiments (Figure S9). Although **AS** was reactive toward H_2_ oxidation as determined by FTIR and EPR spectroscopy (Figures 3B, S7, and S9), we found it to be catalytically inactive under the employed PFE conditions, as reflected in negligible current densities at low and high potentials (Figure S14A). The **A137C** variant also demonstrated a large reduction in both oxidative and reductive currents relative to those of **WT**. However, the currents observed for **A137C** did increase above the blank measurements (Figure S14B).

As previously observed for **WT**,^17^ the proton reduction currents of **CM** and **PTP** do not plateau but rather increase with the driving force, a behavior that is commonly attributed to random binding of the enzymes to the electrode surface and therefore dispersion of interfacial electron transfer rates.^55^ This rate dispersion makes an exact determination of the catalytic onset potential challenging, but it is evident that both **CM** and **PTP** maintain a substantial potential window around *E°*′(2H^+^/H_2_) devoid of significant current (Figure 4A, inset). The irreversible catalytic behavior of all *Tam*HydS variants is in stark contrast to prototypical [FeFe] hydrogenases, which with few exceptions operate with negligible overpotential requirements in both directions (η ≈ 0 mV).^17,37^ Earlier PFE studies of **WT** have confirmed that this is an intrinsic property of the enzyme and not due to slow interfacial electron transfer.^37^ This is further underscored under the mediated electron transfer (MET) conditions. The addition of methyl viologen (MV) to the electrolyte enhanced the proton reduction currents significantly (Figure S15). However, it did not remove the irreversible nature of the catalysis. Instead, the relative rate enhancement increased with an increasing driving force. When comparing currents recorded during MET and DET at pH 6.8, a 3-fold increase in relative rate enhancement for **WT** was observed at −0.60 V that increased to 4-fold at −0.65 V vs NHE. In the case of **CM**, this potential-dependent rate enhancement was even more pronounced: the addition of MV resulted in a 7-fold increase of the current at −0.60 V, while a 12-fold increase was observed at −0.65 V vs NHE.

The redox mediator effect reveals that the maximum currents are limited by electron transfer. The MET data further shows that facilitating interfacial electron transfer through the addition of MV does not fully remove the dependence of the catalytic currents on the overpotential.^56^ The structural rationale behind this irreversible catalytic behavior remains to be fully elucidated, and it is evidently not alleviated in either **CM** or **PTP**. Moreover, the difference in relative rate enhancement as a function of potential when comparing **CM** and **WT** under conditions of MET suggests a higher possible catalytic rate for **CM** given conditions of a sufficiently strong driving force.

The specific activities for H_2_ oxidation and proton reduction were determined in solution assays (Figure 4B). H_2_ oxidation rates were based on the initial rate of change (*V*_0_) determined through colorimetric assays. The assays were performed at 25 °C under anaerobic conditions following injection of H_2_-saturated buffer to a pH 6.8 solution of the respective enzyme. Considering the overpotential requirement of the variants, the effect of driving force was probed using different electron acceptors, *i.e*., benzyl viologen (BV, *E*^0′^ = −0.359 V vs NHE,^57^Figure S16) and methylene blue (MB, *E*^0′^ = 0.008 V vs NHE,^57^Figure S17). In parallel, H_2_ evolution activities were determined via gas-chromatography assays.^58,59^ The amount of H_2_ in the reaction mixture headspace was measured after 15 min of incubation at pH 6.8 in the presence of methyl viologen (MV, *E*^0′^ = −0.446 V vs NHE^57^) as electron mediator and sodium dithionite as a sacrificial electron donor. The measured specific activities (units per milligram) are summarized in Table S5, and one unit of activity indicates 1 μmol of H_2_ oxidized or evolved per minute under the employed assay conditions.

At low driving force, when BV was used as the electron acceptor, the specific activities for H_2_ oxidation displayed by **PTP** (2.43 ± 0.52 U/mg) and **CM** (1.67 ± 0.03 U/mg) are comparable to that of **WT** (1.95 ± 0.27 U/mg). However, the H_2_ oxidation capacity of **WT** substantially increased (22.1 ± 0.8 U/mg) when MB was used as an electron acceptor in the solution assay, outcompeting **PTP** and **CM** (3.70 ± 0.29 and 2.88 ± 0.89 U/mg, respectively). The deviating activities between the variants with an increased driving force for oxidation are in good agreement with the effect of overpotential on H_2_ oxidation current densities measured in PFE (Figure 4A).

A distinctly different trend was observed for the H_2_ evolution rates. One-way ANOVA (Analysis of Variance) with Tukey’s Honestly Significant Difference Test^60^ was used to compare the means of the variants including **WT**. At the *p* = 0.05 level, there is no significant difference in mean H_2_ evolution activity rates between **WT** and **PTP**, displaying specific activities of 0.25 ± 0.03 and 0.60 ± 0.01 U/mg, respectively. As expected from the PFE experiments, **AS** displayed negligible activities in both oxidation and reduction assays, while **A137C** showed only ≈10 and 25% residual activity for H_2_ evolution and oxidation, respectively (Figure 4B). Lastly, **CM** increased the activity up to 170-fold versus **WT**, with **CM** having a specific activity for H_2_ evolution of 36.2 ± 1.8 U/mg. This improved capacity for H_2_ production in **CM** is in line with the PFE experiments, where **CM** showed a higher relative current enhancement compared to **WT** under MET conditions (Figure S15).

### Reactivity toward O_2_ of Variants

**Figure 5:**
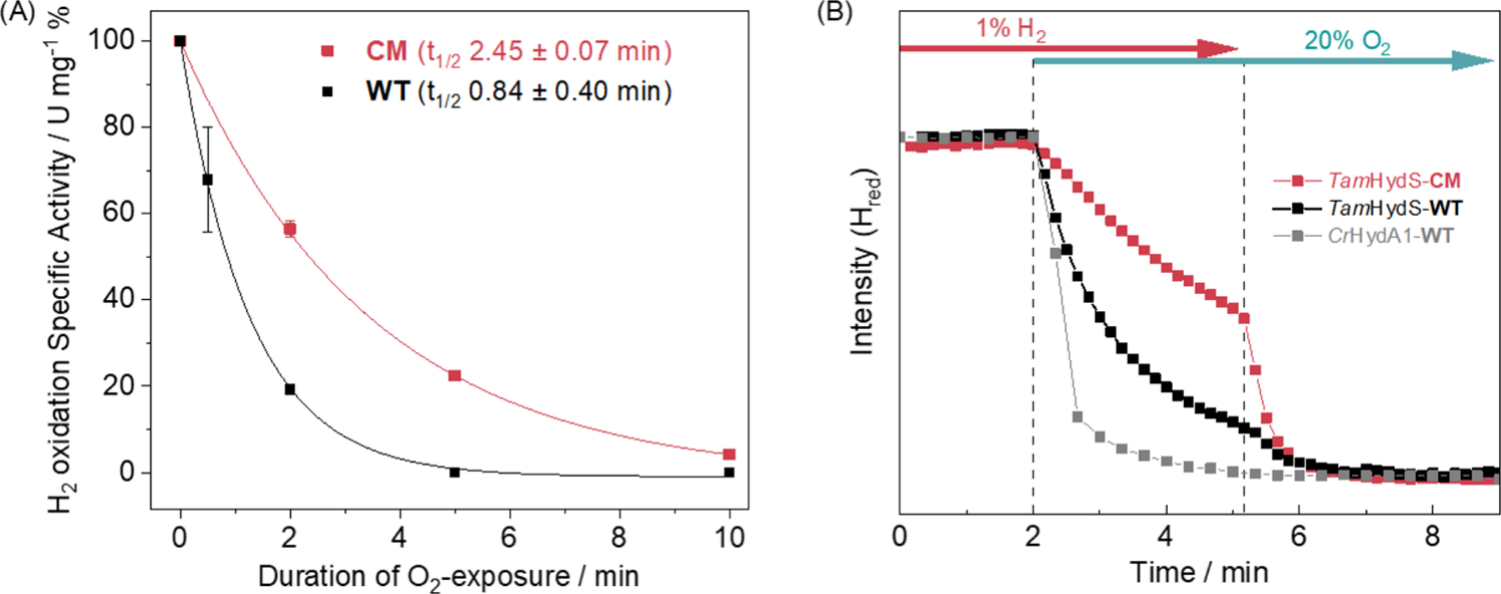
Probing O_2_ sensitivity of the *Tam*HydS variants via enzymatic assays and spectroscopy. (A) Stability toward air was monitored via H_2_ oxidation assays (Figure S16). The relative specific activities of **WT** (black) and **CM** (red) were measured following incubation under air (approximately 21% O_2_) for 0.5–10 min. The data agrees with exponential decay fits (solid lines). The half-life values (*t*_1/2_) extracted from the single exponential decay curves are in parentheses. Data are presented as a mean of 2–3 technical replicates, with error bars showing SEM. (B) Prolonged O_2_ tolerance in **CM (**red) compared to **WT** (black) and *Cr*HydA1 (gray) is reproduced by *in situ* ATR-FTIR spectroscopy following the decay of the H_red_ state over time. The presence of 1% H_2_ in the gas phase stabilizes H_red_ and prolongs the deactivation of *Tam*HydS, which is most clearly observed with **CM**. A similar protection was observed in CO inhibition (Figures S8–S10).

The sluggish reactivity of **CM** toward CO suggests that enhanced activity can be achieved without negatively impacting inhibitor tolerance. To further probe its inhibition properties, the susceptibility of **CM** toward O_2_ was analyzed. The effect of O_2_ on catalytic capacity was tested by incubating dilute aliquots (50 μL, 2 μM enzyme) of **WT** and **CM** under air, in the absence of any reductant in the buffer. Samples were treated with air for 0.5–10 min before being flushed with argon and returned to the glovebox. Subsequently, residual H_2_ oxidation activity was determined by using the BV assay described above. As shown in Figure 5A, O_2_ caused irreversible inactivation of the enzymes, and the process followed apparent first-order kinetics. A half-life time (*t*_1/2_) of 0.84 ± 0.40 min was determined for **WT**. **CM** substantially improved the O_2_ tolerance of **WT**, showing an approximate 3-fold extension of the half-life (*t*_1/2_ = 2.45 ± 0.07 min). **PTP** displayed intermediate O_2_ tolerance, with *t*_1/2_ = 1.45 ± 0.20 min (Figure S18).

The increased O_2_ tolerance of **CM** was verified by ATR-FTIR spectroscopy. Samples of **WT** and **CM** were pre-equilibrated in the H_red_ state under a 1% H_2_ atmosphere. The subsequent introduction of 20% O_2_ into the atmosphere resulted in a loss of the H_red_ population on a minute time-scale for both the **WT** and **CM**. The rate of H_red_ loss increased as the H_2_ component of the atmosphere was removed. As seen in Figure 5B, already the **WT** was more stable than the prototypical *Cr*HydA1 [FeFe] hydrogenase, and this stability was further improved in **CM**. Monitoring the products of the addition of O_2_ revealed further differences between **WT** and **CM** (Figure S19). In all experiments, H_ox_ is the initial product of O_2_-induced oxidation. However, while **WT** displayed parallel formation of the H_air_ state, reflecting a partially degraded H-cluster,^17^**CM** instead forms **State 1**. The **PTP** variant was also found to adopt **State 1** in the presence of 20% O_2_ albeit from **State 2** rather than H_red_ or H_ox_ (Figure S19).

## Discussion

The results presented in this work describe the spectroscopic, electrochemical, and biochemical features of rationally designed *Tam*HydS variants, providing new insights into how interactions between the protein environment and the H-cluster modulate the overall catalytic performance and inhibitor tolerance of [FeFe] hydrogenase.

Optimizing the active-site environment or the proton-transfer pathway in isolation was not sufficient to improve the catalytic properties of *Tam*HydS. Only when the two structural features of **AS** and **PTP** are combined, a clear synergistic effect is observed resulting in significantly enhanced H_2_ evolution rates in **CM**. The improved capacity for proton reduction was clearly evident in solution assays (Figure 4B) and further supported by PFE through the observed current enhancements under the conditions of MET (Figure S15). The latter observation is important considering the recent report that [FeFe] hydrogenases can be inhibited by oxidation products of NaDT formed in standard hydrogenase solution assays,^61^ as it argues against a rate enhancement originating from improved inhibitor tolerance in **CM**.

The fact that **CM** maintains an irreversible catalytic response with midpoint potentials of the reductive and oxidative catalytic waves similar to those of the **WT** variant (Figure 4A) strongly suggests that the H-cluster reduction potentials remain largely unaltered relative to those of **WT**. This conclusion is supported by a recent study demonstrating that the midpoint potentials are directly related to the redox potentials and p*K*_a_ values of the associated protonation steps at the active site of *Tam*HydS.^52^ Since it is highly unlikely that the redox potentials and p*K*_a_ values both change to compensate for each other’s contribution, other underlying mechanisms should be at play in influencing the catalytic properties of the variants.

A priori, it could be speculated that recreating the sulfur-rich environment of prototypical [FeFe] hydrogenase will alter the electronic structure and geometry of the H-cluster, improving its catalytic properties. However, the **AS** variant shows that this is clearly not sufficient to improve catalysis in the Group D enzyme. A related observation was recently reported for the low-activity Group C enzyme *Tm*HydS, where modifications of amino acids in the active-site pocket did not increase the catalytic rates.^62^ The absence of current in cyclic voltammetry and the lack of detectable activity in solution assays instead provide evidence of the loss of catalytic function with **AS**. The EPR and FTIR spectroscopy data confirm that the H-cluster of **AS** has retained the capacity for H_2_ activation. Instead, we attribute the loss of catalytic function to modifications in the active-site hydrogen-bonding network, due to steric displacements and changes in the electrostatic interactions. Collectively, we expect these changes to cause a disruption of the *Tam*HydS’ native proton-transfer pathway and effectively isolate the H-cluster from the bulk solution.^35^

**Figure 6:**
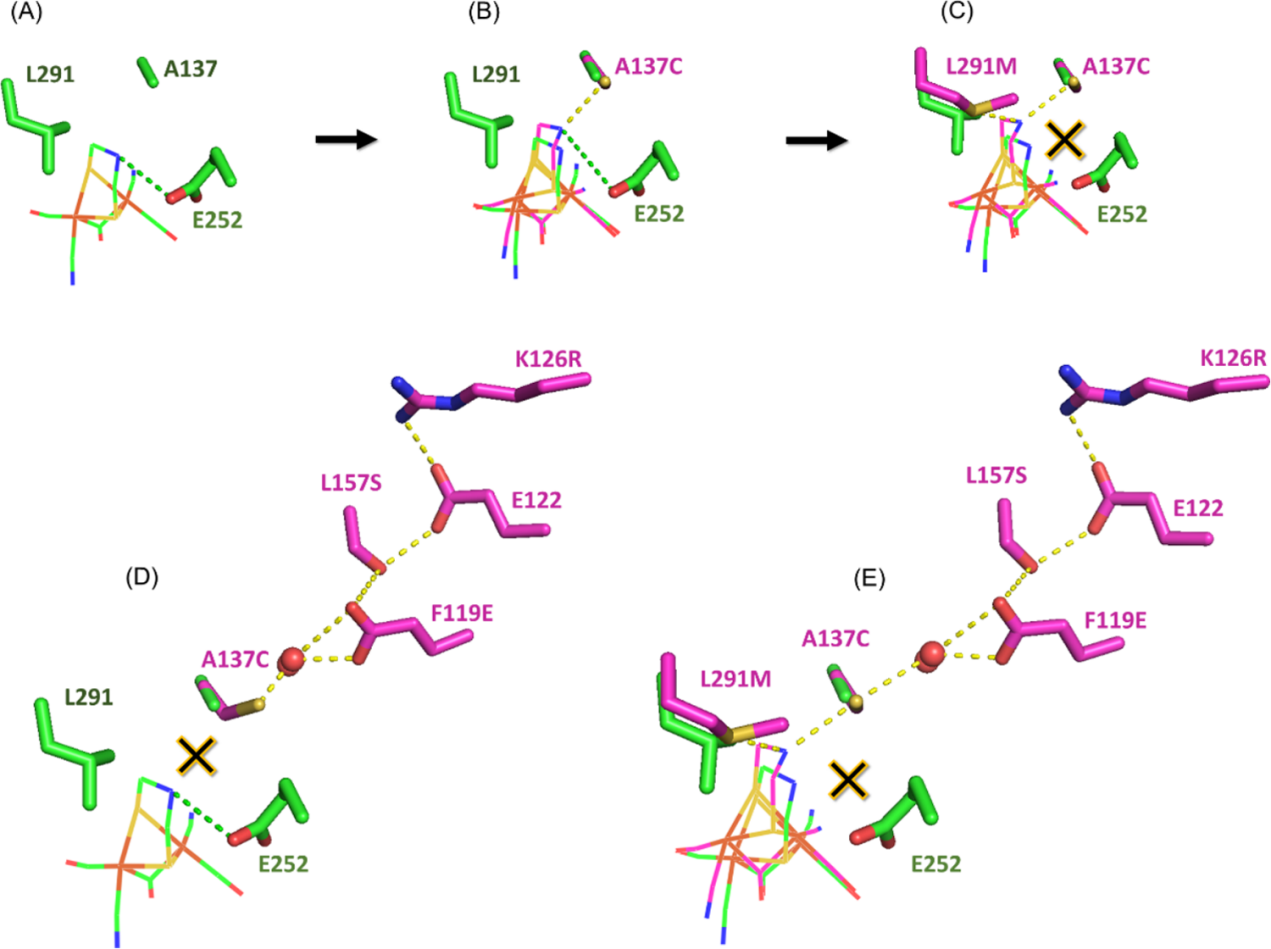
Proposed effect of single-point variant, **A137C**, in isolation and combined with other variants. (A) Homology model of *Tam*HydS highlighting residues L291 and A137 (green sticks), targeted for variation, and the proposed native proton relay residue of *Tam*HydS E252 (green stick) located ≈3.4 Å from the amine bridgehead of ADT (green dashed lines).^35^ The H-cluster (diiron subsite is only shown for clarity) is depicted as a wire with C: green, O: red, N: blue, S: yellow, and Fe: orange. (B) **A137C**, as a lone variant, where the position of the A137C residue (pink stick) corresponds to C299_*Cp*I_ (the position of all residues labeling in pink are based on the crystal structure of *Cp*I, PDB ID 4XDC(18)). The introduced cysteine potentially interacts with the amine via electrostatic interactions (yellow dashed lines) or H-bonding (3.5 Å).^18,21^ The proposed modified position of the H-cluster in this variant is depicted as a wire with C: pink, O: red, N: blue, S group: yellow, and Fe: orange. (C) In the triple variant **AS**, A137C is combined with L291M (and S191M, not shown). The L291M (pink stick) can also interact with the amine bridgehead potentially via H-bonding (3.6–3.7 Å) or increase flexibility due to decrease of steric hindrance.^18,21^ This dual H-bonding interaction scheme is proposed to be detrimental due to complete disconnect of the ADT-amine from the *Tam*HydS’ native proton-transfer pathway. (D) In the quadruple variant **PTP**, A137C is combined with other residues (pink sticks), steering A137C away from the amine bridgehead (note that here the A137C residue deviates from the crystal structure of *Cp*I as it is manually shifted to illustrate the proposed effect). This shift reduces the interaction of A137C with the ADT-amine, allowing for continuous operation of the native proton-transfer pathway of **WT**. (E) In the sextuple variant **CM**, reintroducing L291M enables A137C to reconnect with the amine bridgehead and now also connects the ADT-amine to the improved PTP.

The characterization of the single-point variant **A137C** allows us to propose a model for how the triple variant **AS** completely abolished catalytic activity. A137_*Tam*HydS_ corresponds to the active-site cysteine of Group A (*i.e*., C299_*Cp*I_, C169_*Cr*HydA1_, C298_*Ca*HydA1_), which is known to be essential for coupling the H-cluster to the proton-transfer pathway, and likely fine-tunes the spatial positioning of the amine bridgehead of the ADT ligand through H-bonding.^21,48^ Assuming that **A137C** behaves like the active-site cysteine in Group A enzymes, we can consider two different scenarios: when it is acting alone or in conjunction with the methionines in **AS**. A structural visualization of **A137C** and its potential effect on the positioning of the H-cluster, independently and when combined with other variations, is depicted in Figure 6.

In *Tam*HydS and other Group D [FeFe] hydrogenases, a glutamate positioned at E252_*Tam*HydS_ has been proposed to replace the active-site cysteine of Group A enzymes (C299_*Cp*I_) as the proton relay residue (Figure 6A).^35^ As a standalone variant, **A137C** may interact with ADT-amine, likely via H-bonding (Figure 6B). Previous reports indicate longer than expected H-bonding distances between the cysteine and the amine bridgehead: 3.5 and 3.2 Å for *Cp*I^7,18^ and *Dd*H,^8^ respectively, although computational studies suggest these distances could be shorter (2.1 Å).^63^ We propose that introduction of this cysteine into the active site of *Tam*HydS causes an elongation of the distance between the ADT-amine and the native proton relay (E252_*Tam*HydS_) but not enough to completely sever their connection and entirely abolish catalytic activity (Figure 6B).

Introducing the methionine L291M (corresponding to M497_*Cp*I_ and M415_*Cr*HydA1_) in triple-variant **AS** opens up the possibility of another H-bonding interaction to the ADT-amine (Figure 6C). In combination, A137C and L291M may modulate the spatial positioning and proton conducting ability of the ADT-amine (via NH–S or N–HS bonds) and contribute to the increased flexibility of the active site (Figure 6C). This dual interaction negatively impacts the catalysis of *Tam*HydS, as evidenced by the observed loss of catalytic activity in **AS**, most likely due to the disconnect of the amine bridgehead from the native proton-transfer pathway. The potential influence of S191M (corresponding to M353_*Cp*I_ and M223_*Cr*HydA1_) has not been probed in detail here, leaving its role in tuning the H-cluster somewhat ambiguous. However, the methionine in this position (Figure 1B) can interact electrostatically with the bridging CO, and it has been previously suggested that this interaction is crucial for achieving high turnover rates, adjusting affinity for H_2_, and modulating H-cluster reactivity.^21,38^

More broadly, considering the highly conserved nature of the active-site methionines in Group A [FeFe] hydrogenase, H-bonding is unlikely to be their sole role. Moreover, the distances observed by crystallography in Group A [FeFe] hydrogenases between the ADT-interacting methionine (M497_*Cp*I_) sulfur and the ADT-amine are again somewhat longer than would be expected for a H-bonding interaction, 3.6–3.7 Å for *Cp*I^7,18^ and 3.9 Å for *Dd*H.^8^ Instead, the relatively soft nature of the methionine sulfur is likely to further allow the ADT ligand to optimize its position relative to Fe_d_ and active-site cysteine. The side chain of methionine is known for its significant conformational adaptability, due to the length and low rotational energy barrier of the C_methyl_–S bond,^64^ while leucine, albeit having a fairly large side chain, is still less flexible than methionine due to higher rotational energy barriers. For Group A [FeFe] hydrogenases, it has been proposed that M497_*Cp*I_ may act as a “cushion” between the H-cluster and the peptide structure by absorbing the impact of their relative movements.^21^ The specific substitution of methionine with leucine in the putatively sensory *Tam*HydS **WT** raises intriguing questions. We hypothesize that the increased rigidity conferred by leucine might enhance detection of external stimuli (*e.g*., H_2_ pressure) and ensure consistent signaling. Conversely, catalytic enzymes might require a more flexible active site to accommodate substrates more effectively and allow for rapid transitions between conformational states.^65^ In another sensory hydrogenase, Group C *Tm*HydS, the methionine in this position is replaced by serine.^62^ While serine can form H-bonds, it is generally considered to have less conformational flexibility than methionine. Similar to L291M, the methionine side chain of S191M can also introduce additional flexibility within the active-site environment. Prior studies on Group A have shown that replacing this methionine (M353_*Cp*I_) with a more rigid leucine led to reduced activities compared to wild-type enzymes, though not as severely as variations in the two residues interacting with the amine bridgehead (*i.e*., C299_*Cp*I_ and M497_*Cp*I_).^38^ Exploring the balance between rigidity and flexibility in sensory and catalytic metalloenzymes and their impact on the physiological function presents an interesting point of further investigation.

Moving beyond the immediate active-site environment, introducing the Group A-type proton-transfer pathway could accelerate catalysis through a faster long-range proton transfer. However, no enhanced catalytic activity was observed in the **PTP** variant. We speculate that the H-cluster is not positioned to benefit from the engineered proton trajectory as long as the active-site methionines interacting with the H-cluster are missing (Figure 6D). When A137C is not paired with L291M but instead linked with other residues more distant from the H-cluster, the interaction of A137C with the amine bridgehead is likely reduced due to redirection of the cysteine thiol by electrostatic or H-bonding interactions with other PTP residues or water molecule(s) (Figure 6D). As a consequence, the incorporation of additional PTP residues does not result in faster catalysis but alleviates the negative effects of **A137C**, as the variant enables the native proton-transfer pathway via E252_*Tam*HydS_ to function continuously, thereby, preserving the catalytic activity of **WT**.

With regard to **PTP**, it is also noteworthy that the FTIR spectra of this variant is dominated by **State 2** under a H_2_ atmosphere, in contrast to **WT** and **CM** that instead display primarily H_red_ with a small population of **State 2** only discernible by EPR spectroscopy under equivalent conditions. The single-point variant **A137C** also exhibits a clearly discernible population of **State 2**, albeit H_red_ also forms upon H_2_ treatment (Figure 3A). The origin of this difference in the H-cluster state accumulation has not been identified. However, considering that **PTP** displays similar H_2_ formation rates as **WT**, it suggests that **State 2** is catalytically competent.

Considering the reactivity of the **PTP** and **AS** variants, the increased H_2_ formation rates observed for **CM** highlight the influence of the protein matrix on H-cluster reactivity, as well as the complexity associated with macromolecular catalyst design. The rate enhancement in **CM** is in good agreement with the notion that the catalytic performance of **WT***Tam*HydS is limited by long-range substrate (proton) transport during H_2_ formation. However, the faster proton-transfer kinetics provided by the Group A-type proton-transfer pathway must evidently be coupled with fine-tuning of the active-site structure for the rate enhancement to become apparent (Figure 6E). Following the reasoning above, the two methionine residues would then serve to reposition the H-cluster so as to connect it to the alternative “faster” proton-transfer pathway. Nevertheless, **CM** continues to display overpotential requirements that are significantly larger than those of Group A enzymes. This means that although a 170-fold increase in the H_2_ evolution rate was observed for **CM**, the specific activities as determined by the MV-based assay possibly underestimate the maximum rate. Indeed, the CV measurements with MV as a mediator further reveal that the proton reduction rate enhancement in **CM** is dependent on the applied overpotential. While the oxidation assay could be readily adapted to increase the driving force (*i.e*., employing benzyl viologen or methylene blue as the electron acceptor), the reduction assay is limited by the lack of suitable viologen redox mediators with reduction potentials below that of MV. The driving force dependence on H_2_ evolution rates in solution presents an important line of inquiry moving forward.

A key feature of *Tam*HydS is its relatively high tolerance toward inhibitors, as compared to previously studied prototypical [FeFe] hydrogenases.^17^ Efforts to improve catalytic features and O_2_ tolerance of hydrogenases to O_2_, based on proposed molecular mechanisms of proton, gas, and electron transfer, are still limited. Most protein engineering studies to shift the catalytic bias toward proton reduction and increase O_2_ tolerance have been performed on [NiFe] hydrogenases, and some success has been achieved.^66−73^ Parallel attempts of optimizing Group A [FeFe] hydrogenases have so far proven challenging,^23,74^ although it has been shown that there is no strict inverse correlation between O_2_ tolerance and high catalytic rates.^75^ In line with the latter observation, **PTP** increased the level of tolerance of O_2_ with retained H_2_ production activities relative to **WT**, while **CM** provides a striking example that rates can be improved concomitantly with inhibitor tolerance. Three main mechanisms for O_2_ protection have been observed in hydrogenases, including (i) complete reduction of O_2_ to benign H_2_O, avoiding the formation of reactive oxygen species (ROS);^42,76,77^ (ii) restricting access of inhibitors via proteinaceous gas filters;^78,79^ and (iii) external ligand binding, such as the thiol-inhibited state H_inact_^44,80,81^ or hydroxide-inhibited Ni–A and Ni–B states of [NiFe] hydrogenase.^82^ The slow reaction with CO observed for **CM** could be due to more limited gas access, arguing for inhibition protection via a gas filter effect in the variant. However, our findings suggest a further protective mechanism that involves the binding of an external ligand, as **CM** and **PTP** exhibited FTIR spectra akin to those of H_inact_, *i.e*., **State 1** forming upon exposure to O_2_ (Figure S19, Supporting Note 1).^35^ We speculate that the earlier proposed aqua (or hydroxido) ligand binding in **State 1** can protect **CM** and **PTP** against O_2_ attack,^35^ similar to [NiFe] hydrogenase.^82^ It follows that **State 1** would be an inhibited state, and the formation of **State 1** upon oxidation could also rationalize the oxidative inactivation observed for **CM** and **PTP** in PFE experiments (Figure 4A).

## Conclusions

This study demonstrates how design aspects of two phylogenetic groups can be employed for improving the performance of a macromolecular catalyst through rational design. It shows the importance of exploring a broader range of [FeFe] hydrogenases to probe the impact of different protein structural features on H-cluster reactivity. By extension, these efforts will also provide new paths toward metalloenzyme engineering and alternative model systems for biomimetic synthetic catalysts. More specifically, the current study provides gain-of-function support for both the proposed proton-transfer pathway of prototypical [FeFe] hydrogenase and the importance of the sulfur-rich active-site canopy for fast catalysis. We hypothesize that the observed rate enhancement in **CM** arises from optimized coupling between the engineered faster proton-transfer pathway and the H-cluster, which relies on the precise positioning of the central proton relay cysteine as well as the [2Fe]_H_ subsite for optimal engagement with ADT-amine. This interaction is critically contingent on the presence of another sulfur-containing residue, methionine, that also interacts with the amine bridgehead. While the role of the second methionine, positioned on the opposite side of [2Fe]_H_ close to the μCO ligand, remains to be fully elucidated, it is conceivable that the introduced sulfur-rich residues collectively result in a relatively more flexible active-site environment for the H-cluster, allowing faster conformational state transitions. We note that the identified coupled effects between structural features, *i.e*., the proton-transfer pathway and active-site environment, would be difficult to identify via classical loss-of-function studies. The factors that govern the irreversible catalytic response of *Tam*HydS remain elusive, and solving this issue represents an intriguing molecular design challenge. Nonetheless, the study highlights that activity rates, overpotential, and susceptibility to gaseous inhibitors are decoupled and thus can be tuned separately. Indeed, [FeFe] hydrogenases can evidently be tailor-made to become faster and more robust catalysts through rational design. Lastly, it highlights a critical design challenge with regard to the current trend of immobilizing molecular catalysts in larger (electrocatalytic) polymers, as the catalyst positioning relative to the proton (substrate) delivery pathway needs to be optimized in tandem.

## Supporting Information Available

Experimental procedures, Alphafold model of *Tam*HydS, AGE/PAGE gels, Fe/protein content, additional EPR and FTIR data, PFE data, absorbance traces for reconstitution and solution assays, and an extended discussion on the properties of **State 1** and **State 2** (PDF)
