# Supplementary material for: Probing the Influence of the Protein Scaffold on H-Cluster Reactivity via Gain-of-Function Studies—Improved H2 Evolution and O2 Tolerance through Rational Design of [FeFe] Hydrogenase
Source: J Am Chem Soc. 2025 Jan 27;147(5):4654–66. doi: 10.1021/jacs.4c17364 (PMC11803613; doi:10.1021/jacs.4c17364)
Supplement: Supplementary file 1 — ja4c17364_si_001.pdf [file ja4c17364_si_001.pdf]

Supporting information

Probing the influence of the protein scaffold on H-cluster reactivity via gain-of-function studies - Improved H_2 evolution and O_2 tolerance through rational design of [FeFe] hydrogenase

Princess R. Cabotaje[1], Alina Sekretareva[1], Moritz Senger[1,2], Ping Huang[1], Kaija Walter[1], Holly J. Redman[1], Nicholas Croy[1], Sven T. Stripp[3,*], Henrik Land[1,*], Gustav Berggren[1,*]

[1] Molecular Biomimetics, Department of Chemistry, Ångström Laboratory, Uppsala University, Box 523, Uppsala, SE-75120, Sweden
[2] Biochemistry, Department of Chemistry, Biomedical Centre, Uppsala University, Uppsala, SE-75120, Sweden
[3] Spectroscopy and Biocatalysis, Institute of Chemistry, Universität Potsdam, Potsdam, D-14476, Germany

* gustav.berggren@kemi.uu.se, henrik.land@kemi.uu.se, sven.stripp@uni-potsdam.de

Experimental Procedures 4
General 4
Generation of *Tam HydS* variants........ 4
Aerobic expression and anaerobic purification of apo-*Tam*HydS variants 5
Semi-enzymatic reconstitution of FeS clusters 6
Generation of holo-*Tam HydS* variants 6
Protein film electrochemistry........ 6
In vitro H_2-evolution and -oxidation Assays 7
H_2-evolution........ 7
H_2-oxidation........ 8
ATR FTIR spectroscopy 8
EPR spectroscopy 8
Supplementary figures and tables........ 9
Table S1. Summary of *Tam*HydS mutations. 9
Figure S1. Comparison of *Tam*HydS Alphafold and homology models 10
Figure S2. AGE gels of PCR amplicons. 11
Figure S3. PAGE gels of expressed and purified variants. 12
Table S2. Fe:protein ratio before and after reconstitution, as well as after activation with $[2Fe]^{ADT}$. 13
Figure S4. Semi-enzymatic reconstitution of the variants' [4Fe-4S] clusters. 14
Figure S5. EPR spectra of visualizing the $[4Fe-4S]^+$ clusters of NaDT-reduced apo-*Tam*HydS 15
Table S3. Tabulated g-values of selected states reported for the H-cluster. 16
Figure S6. Temperature and power dependence studies of H_{ox}-CO signals 17
Figure S7. EPR spectra of H_2-reduced holo-*Tam*HydS WT and variants. 18
Supporting Note 1 19
Figure S8. Spectral characterization 20
Table S4. Summary of FTIR bands vs Group A. 21
Figure S9. Kinetics of state conversions. 22
Figure S10. Reaction with H_2 and CO monitored in FTIR. 23
Figure S11. Second scan CV traces of *Tam*HydS and variants. 24
Figure S12. Changes in current densities with scan number. 25
Table S5. Tabulated specific activities 26
Figure S13. Chronoamperometry measurements 27
Figure S14. A. Cyclic voltammograms of AS and A137C 28
Figure S15. DET vs MET electrochemistry experiments 29

Figure S16. Reduction of BV^{2+} → $BV^{•+}$ 30
Figure S17. Reduction of MB^{+} → LMB 31
Figure S18. Exponential decay curves based on *in vitro* O_2-tolerance tests 32
Figure S19. Monitoring O_2 products WT vs CM vs PTP in FTIR. 33
Figure S20. Calibration curves of the gas chromatograph 34
References 35

Experimental Procedures

General

All chemicals were purchased from Sigma-Aldrich or VWR and used as received unless otherwise stated. Protein expression was analyzed by 12% SDS-PAGE mini gels in a BIO-RAD Mini PROTEAN® system. The proteins were stained with Page Blue protein staining solution (Thermo Fisher Scientific) according to the supplier instructions. All anaerobic work was performed in an MBRAUN glovebox ([O2] < 10 ppm). The $[2Fe]^{ADT}$ was synthesized following literature protocols with minor modifications and verified by FTIR spectroscopy.[1] UV-Vis spectra were obtained using an AvaSpec-ULS2048-USB2-UA-50: Avantes Fiber Optic UV/VIS/NIR spectrometer using a 1-mm pathlength absorption cuvette for reconstitution and 2-mm/1cm plastic cuvette for biochemical *in vitro* assays.

Generation of *Tam HydS* variants

The gene encoding for *Tam*HydS was synthesized with a C-terminal Strep-Tag^{II} sequence and cloned in pET-11a(+) by Genscript® using restriction sites *NdeI* and *BamHI* following codon optimization for expression in *Escherichia coli*. The plasmid also contained a lac operator that initiates transcription in the host cell upon the addition of isopropyl-β-D-1-thiogalactopyranoside (IPTG). All primers designed to introduce the site-directed mutation were synthesized and purified by Eurofins Genomics (Ebersberg, Germany). The melting temperatures (Tm) were obtained using the calculator developed by ThermoFisher Scientific (optimum range: 65 to 72°C). To avoid the formation of dimers and hairpin loops, ΔG values for secondary structures were calculated by Integrated DNA Technologies (IDT) OligoAnalyzer (0 to −3 kcal/mole for optimal design). Parental plasmid harboring the wild-type (**WT**) gene was amplified by PCR with Phusion® High-Fidelity to generate the constructs for the expression of four site-directed mutagenesis (SDM) variants (**AS**, **PTP**, **CM** and **A137C**) using the following optimized primers:

**Mutation**			**Primer Sequence**		**Tm (°C)**
**A137C**	A137C_forward	5'	**GCAGCTGCTGCCCGGCGG**	3'	69.4
	A137C_reverse	5'	**CGGGCAGCAGCTGCTAATAACCGG**	3'	68.4
**F119E**	F119E_forward	5'	**GCAAGGAAACCCGTGAAGCGC**	3'	66.9
	F119E_reverse	5'	**CGGGTTTCCTTGCTAACAATCTCCG**	3'	66.0
**L157S**	L157S_forward	5'	**CAACCTGAGCGATATCTGCAGCCC**	3'	65.9
	L157S_reverse	5'	**CAGATATCGCTCAGGTTGTCAATCAGGC**	3'	66.2
**K126R**	K126R_forward	5'	**GGCGCGTGGTAACCTGAAGAAACCG**	3'	68.3
	K126R_reverse	5'	**GGTTACCACGCGCCAGCGCTTC**	3'	68.7
**S191M**	S191M_forward	5'	**GTTCTTTATTATGCCGTGCGCGG**	3'	64.8
**L291M**	L291M_reverse	5'	**CACGCCATGCCTTCAAAGAAATCC**	3'	67.3

The 50 µl PCR mixture was carried out with 10 ng plasmid template, 1 mM dNTPs, 0.5 µM each for the forward and reverse primers, 1 unit (U) of Phusion High-Fidelity (HF) polymerase, in 1x HF buffer with varying DMSO concentrations from 0 to 8%. The extension reaction was initiated by pre-heating the reaction mixture to 98°C for 30 s; followed by 30 cycles of (1) 98°C for 30 s, (2) Ta = $[(Tm_{forward} + Tm_{reverse})/2]$ - 2°C for 30 s, and (3) 72°C for 3:30 min according to the length of template; and then completed by incubating at 72°C for 5 min. The PCR product was then digested with *DpnI* restriction enzyme to eliminate parental, methylated DNA under the following conditions: 37°C for 2 h; 80°C for 5 min; and stored at 12°C until the amplification products were evaluated by agarose gel electrophoresis.

An aliquot of 1 µl PCR product was transformed into chemically competent *E. coli* DH5α cells using heat shock at 42°C. Single colonies were transferred on agar plates supplemented with 100 µg/mL ampicillin. Ampicillin-resistant colonies were grown overnight in 5 mL LB medium with the same antibiotic concentration at 37°C. Expression constructs were extracted from the small-scale overnight culture using GenElute™ Plasmid Miniprep Kit, and gene integrity was verified via sequencing by Eurofins Genomics. The verified plasmid was then used as the template for the subsequent PCR step to introduce another mutation under the same conditions as described except the annealing temperature (T_a) changes, depending on the Tms of the primer pair.

For the construction of the **AS** variant, template plasmid with a verified A137C mutation, and around 0.5 µg of megaprimer, generated from S191M_forward and L291M_reverse primers, were used in an additional PCR step to mutate two sites at once using the same conditions as described except the annealing temperature (Ta) was set to 55°C.[2]

The **AS** variant harbored three mutations: A137C, S191M, and L291M; the **PTP** variant had four mutations: A137C, F119E, L157S, K126R; and the **CM** variant had six mutations: A137C, S191M, L291M, F119E, L157S, and K126R.

Aerobic expression and anaerobic purification of apo-*Tam*HydS variants

Expression constructs with verified sequences were retransformed in chemically competent *E. coli* BL21(DE3) cells to express the *apo*-forms of *Tam*HydS (**WT** and variants) lacking the diiron subsite of the H-cluster. Starting cultures were grown overnight in 10 mL LB medium containing 100 µg/mL ampicillin at 37°C. These cultures were subsequently used to inoculate 1 L of M9 medium (22 mM Na_2HPO_4, 22 mM KH_2PO_4, 85 mM NaCl, 18 mM NH_4Cl, 0.2 mM $MgSO_4$, 0.1 mM $CaCl_2$, 0.4% (v/v) glucose) containing 100 µg/mL ampicillin. Cultures were grown at 37°C and 150 rpm until an optical density (OD_{600}) of approximately 0.4 to 0.6 was reached. Protein expression was induced by the addition of 0.1 mM $FeSO_4$ and 1 mM IPTG. Induced cultures were incubated at 20°C and 150 rpm for approximately 16 h. Cells were thereafter harvested by centrifugation in a Beckman Coulter Avanti J-25 centrifuge (5,000 rpm/4,424 x g, 10 min).

All subsequent operations were carried out under anaerobic conditions in the glovebox to prevent hydrogenase inactivation by atmospheric oxygen. The cell pellet was resuspended in 100 mM Tris-HCl pH 8.0, with NaCl (150 mM), $MgCl_2$ (10 mM), lysozyme from chicken egg white (1 mg/mL), DNAse I from bovine pancreas (0.05 mg/mL), RNAse A from bovine pancreas (0.05 mg/mL), and a tablet of cOmplete™ EDTA-free protease inhibitor cocktail, and was incubated inside the glovebox for 30 min. Cell lysis was performed by three cycles of freezing/thawing in liquid N_2. Cell debris was removed by centrifugation in a Beckman Coulter Optima L-90K Ultracentrifuge (55,000 rpm/222,592

x g, 60 min). The supernatant was collected and filtered (0.45 µm syringe filter) before being loaded on a StrepTrap™ HP (GE Healthcare) affinity column using a BioLogic DuoFlow™ FPLC system (Bio-Rad) and purified according to the manufacturer´s instructions. Products eluted by 2.5 mM d-desthiobiotin were concentrated using Amicon®Ultra 30 kDa molecular weight cut-off (MWCO) centrifugal filters (Merck Millipore Ltd.). In the case of additional impurities, the Strep-trap purified proteins were further separated using size exclusion chromatography via Superdex™ 200 10/300 GL, equilibrated in 100 mM Tris-HCl, 150 mM NaCl pH 8.0, to acquire the purified protein of interest. Samples were stored anaerobically at −80 °C.

Coomassie-stained SDS-PAGE verified the purity of the 50 kDa isolate alongside a prestained protein ladder. Protein estimations were performed via Bradford assay using bovine serum albumin as a standard.[3] Quantification of Fe-content was performed using a previously reported assay[4] using a commercially available Fe^{2+} standard for AAS TraceCERT (Sigma Aldrich) for the calibration curve.

Semi-enzymatic reconstitution of FeS clusters

A solution of 50 µM apoprotein in 100 mM Tris-HCl, 150 mM NaCl pH 8.0 was incubated with 500 µM dithiothreitol (DTT) under strictly anaerobic conditions for 10 min at room temperature. The iron and sulfur sources were ferrous ammonium sulfate and L-cysteine, respectively, both added in 1.5-fold molar excess to the desired number of Fe-atoms to be added. Reconstitution was initiated by adding a 1% molar equivalent of recombinant cysteine desulfurase (*E. coli* IscS), slowly releasing sulfide *in situ* from cysteine. Reaction mixtures were incubated at room temperature for 2 to 3 h. At the same time, the increase of absorbance around 405 nm was monitored by UV/Vis. The reconstitution process was stopped by running the reaction mixture through a PD-10 column (GE Healthcare), equilibrated in 100 mM Tris-HCl, 150 mM NaCl pH 8.0.

Generation of holo-*Tam HydS* variants

Holo forms of the variants for *in vitro* H_2 evolution/oxidation assays, Protein Film Electrochemistry (PFE), FTIR, and EPR analyses were prepared under strictly anaerobic conditions by mixing reconstituted proteins (50 µM) with sodium dithionite (1 mM, 20x excess) in 100 mM phosphate buffer, pH 6.8 and incubated in room temperature for 10 minutes. Cofactor incorporation started with the addition of $[2Fe]^{ADT}$ (600 µM, 12x excess), and the reaction mixture was incubated for 1 to 2 h. The mixture was loaded onto a PD-10 desalting column (GE Healthcare) equilibrated with 10 mM Tris-HCl, 2 mM sodium dithionite pH 8.0. The sample was concentrated using Amicon®Ultra 30 kDa MWCO centrifugal filters (Merck Millipore Ltd.), aliquoted into PCR tubes, and transferred into airtight serum vials (3-5 µL each) before they were flash-frozen in liquid N_2 and stored at -80°C until further use. Gas-treated holo-variants for EPR analyses were exposed to CO or H_2 for 1 hr, before they were transferred to EPR tubes and flash-frozen in liquid N_2.

Protein film electrochemistry

Protein film electrochemistry experiments were carried out under anaerobic conditions. The three-electrode system was made up of (1) Ag/AgCl (4 M KCl/AgCl) as the reference electrode, (2) a rotating disk 5 mm OD edge-plane pyrolytic graphite (PGE) (epoxy encapsulated) as the working electrode,

and (3) a graphite rod as the counter electrode. The gas-tight glass cell used featured a water jacket for temperature control and a cell gas inlet/outlet for hydrogen flow control.

The buffer used was composed of 5 mM MES, 5 mM CHES, 5 mM HEPES, 5 mM TAPS, 5 mM sodium acetate, with 0.1 M NaCl as supporting electrolyte titrated with HCl to pH 6.0, and purged with N_2 for 3 to 4 hours. The PGE working electrodes were polished with an aqueous slurry of 0.3 µm Al_2O_3 on an alumina pad before bringing them into the glovebox. A temperature of 40°C and pH of 6.0 were used throughout the experiments.

To remove residual O_2 in the PGE electrode, cyclic voltammograms were run at 100 mV/s from -100 to -600 mV (vs. NHE) for 40 scans. The cyclic voltammogram of the blank electrode (no enzyme immobilized) was then recorded at 2 mV/s with the working electrode rotated at 3 krpm.

Polycationic polymyxin B sulfate (10 µL of 0.2 mg/mL) was added onto the deaerated PGE surface before adding 10 uL of 5 µM holo-enzyme. The mixture was left for 10 min for maximal adsorption before the excess solution was removed by pipet. The cell was then saturated with H_2 (1 atm, 20 min) under mildly oxidative potential (-300 mV), with the generated current densities monitored using chronoamperometry. The cyclic voltammogram of the system with the immobilized enzyme was then recorded at 2 mV/s under 1 atm of H_2.

For the chronoamperometry data, the current densities were measured at -100 mV vs. NHE, with 1 atm H_2, and the working electrode rotated at 3 krpm.

For the mediated electron transfer (MET) experiments[5], CVs were collected at 20 mV/s, pH 6.8, 40ºC, under 1 atm Ar, with no rotation of the working electrode (0 rpm). Direct electron transfer (DET) CVs were recorded following enzyme immobilization on the PGE electrode. Mediated electron transfer (MET) CVs were recorded after the addition of 560 µM of the redox mediator methyl viologen (MV). Subsequently, the enzyme was denatured by heating at 90ºC for 30 minutes. The buffer was exchanged with a fresh buffer before recording blank CVs. The last CVs were recorded after 560 µM MV was added into the buffer.

Electrochemical data was acquired using an Eco/Chemie PGSTAT10 and the GPES software (Metrohm/Autolab). Data were analyzed using Origin 8 software. All values are referenced versus NHE.

In vitro H_2-evolution and -oxidation Assays

H_2-evolution: Inside the glovebox filled with Ar, the fully-reconstituted holo-enzyme was diluted to 1 µM in 180 µL phosphate buffer (100 mM, pH 6.8) in an 800 uL crimp top vial and sealed with a natural rubber/clear PTFE septum pre-assembled in an aluminum cap (8 mm). The reaction was initiated with the rapid addition of 10 mM methyl viologen as electron mediator, $MV^{\bullet+} \rightarrow MV^{2+}$ ($E^{0'}$ = –0.446 V vs SHE[6]), and 100 mM sodium dithionite as reducing agent/sacrificial electron donor. The mixture was incubated at 28°C and 120 rpm. After 15 min, a 100 µL aliquot from the headspace (total volume of 620 µL) was injected into a PerkinElmer Clarus 500 gas chromatograph (GC) equipped with a thermal conductivity detector (TCD) and a stainless-steel column packed with Molecular Sieve (60/80 mesh). The operational temperatures of the injection port, the oven, and the detector were 100°C, 80°C, and 100°C, respectively. Argon was used as carrier gas at a flow rate of 35 mL/min. To determine the amount of H_2 associated with the peak area at around 0.4 min in the chromatogram, a calibration curve made up of standard points from 0 to 60% H_2 in the 620 µL headspace was generated (**Figure S20**).

One unit (U) of activity catalyzes 1 μmol of H_2 evolved per min under the indicated assay conditions, whereas specific activity is U per mg of the enzyme.

H_2-oxidation: Two redox mediators were used for measuring hydrogen oxidation: benzyl viologen, $BV^{2+} \rightarrow BV^{\bullet+}$ ($E^{0'}$ = –0.359 V vs SHE[6]), and methylene blue, MB^+ to Leucomethylene Blue, LMB ($E^{0'}$ = 0.008 V vs SHE[6]). The absorbance of the mixture containing <30 μg of holo-enzyme and 1 mM benzyl viologen in H_2-saturated 100 mM phosphate buffer pH 6.8 (≈ 1 mM H_2 in solution) was measured at 550 nm in 1 mL plastic cuvettes. A standard curve was generated to determine the molar extinction coefficient of reduced benzyl viologen (ε_{550}^{red} = 9.12 mM^{-1} cm^{-1}). The BV specific activity (U/mg) was measured by the initial rate of change of absorbance at 550 nm where one unit (U) of activity catalyzes 2 μmol of benzyl viologen reduced per min (1 μmol of H_2 oxidized per min) under the indicated assay conditions. For the O_2-tolerance tests in solution, the holo-variants (<30 μg) were exposed to air (20% O_2) for 0.5–10 mins and after which the samples were flushed with Ar and returned to the glovebox. Residual H_2-oxidation activity per time point was measured using the benzyl viologen assay analogous to how the anaerobically incubated holo-variant was measured. All measurements were done at room temperature, RT approximately 25°C. H_2-saturated 100 mM phosphate buffer (≈ 1 mM H_2 in solution) pH 6.8 was added to a mixture of 30 μM methylene blue (ε_{660}^{ox} = 37.9 mM^{-1} cm^{-1}) and holo-enzyme (<100 μg).[7] The MB specific activity (U/mg) was measured by the initial rate of absorbance at 660 nm where one unit (U) of activity catalyzes 1 μmol of methylene blue reduced per min (2 e^- reduction of MB; 1 μmol of H_2 oxidized per min) under the indicated assay conditions.

ATR FTIR spectroscopy

Fourier-transform infrared (FTIR) spectroscopy was performed in ATR configuration on hydrated films of 1 μl isolated [FeFe] hydrogenase (about 500 μM) in 10 mM Tris, pH 8.0. All experiments were performed inside a Coylab glovebox (N_2 atmosphere with 1–2% H_2). Absorbance spectra were recorded on a Bruker Tensor 27 with a spectral resolution of 2 cm^{-1}, and a varying number of interferometer scans at 80 MHz (time resolution 1–10 s). The spectrometer was equipped with a 3-reflection Si ATR unit (Smiths DuraSamplIR II). All gas titrations were performed according to established protocols.[8]

EPR spectroscopy

X-band EPR measurements were performed on a Bruker ELEXYS E500 spectrometer equipped with a SuperX EPR049 microwave bridge and a cylindrical TE011 ER 4122SHQE cavity in connection with an Oxford Instruments continuous flow cryostat. Measuring temperatures were achieved using liquid helium flow through an ITC 503 temperature controller (Oxford Instruments). The Xepr software package (Bruker) was used for data acquisition and processing. EasySpin software version easyspin-6.0.0-dev.51 was used for spectral simulation and fitting.[9, 10]

Supplementary figures and tables

Table S1. Summary of *Tam*HydS mutations.

Mutant		*Tam* HydS	*Tm* HydS	*Cp* I	mutation
Active site	AS	A137	A131	C299	A137C
		S191	G177	M353	S191M
		L291	S267	M497	L291M
Proton Transfer Pathway	PTP	A137	A131	C299	A137C
		F119	Y116	E279	F119E
		L157	A151	S319	L157S
		K126	H123	R286	K126R
Active Site + Proton Transfer Pathway	CM	A137	A131	C299	A137C
		F119	Y116	E279	F119E
		L157	A151	S319	L157S
		K126	H123	R286	K126R
		S191	G177	M353	S191M
		L291	S267	M497	L291M

Table S1. Summary of *Tam*HydS mutations. AS featured non-conservative exchanges to introduce sulfide groups (A137C, S191M, and L291) near the vicinity of the H-cluster. **PTP** has two non-conservative exchanges (F199E and L157S) and one conservative exchange (K126R). The latter maintains the functional group of the targeted position but can significantly alter the spatial arrangement of the positively charged/basic functional group within the enzyme. **CM** merges the variations in **AS** and **PTP**. The table also shows *Tam*HydS amino acid residues corresponding to that of Group A *Cp*I and Group C *Tm*HydS.

Figure S1. Comparison of *Tam*HydS Alphafold and homology models

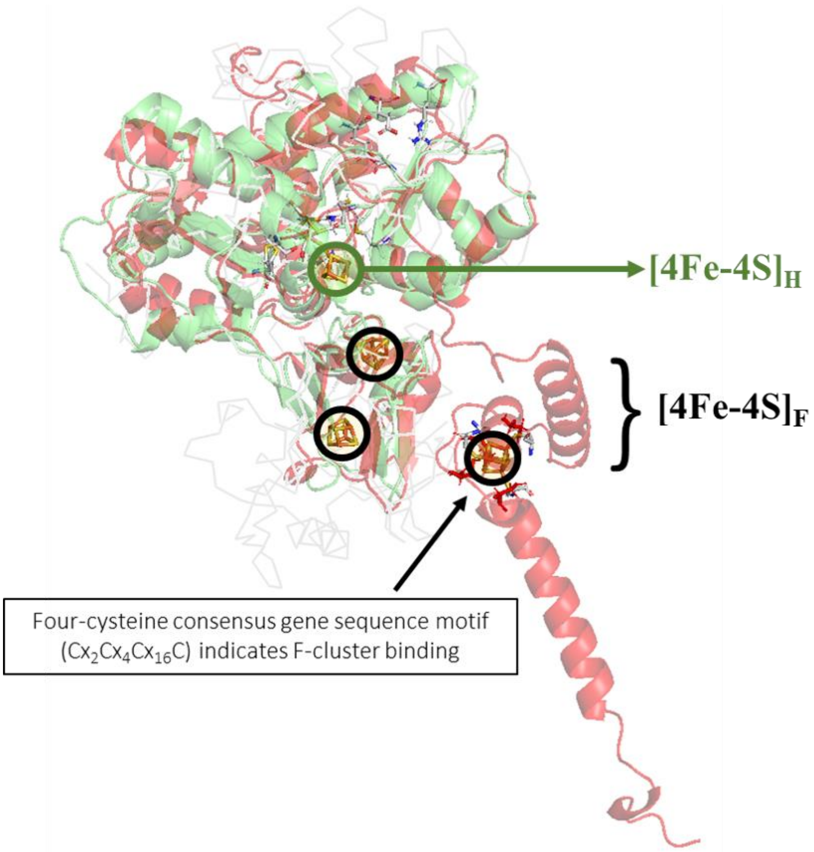

Figure S1. Comparison of the *Tam*HydS Alphafold model (red cartoon) and YASARA-generated homology model of *Tam*HydS[11] (green cartoon). Both models are aligned with the crystal structure of *Cp*I (PDB ID 4XDC[12]). The Root Mean Square Deviation (RMSD) values, which measure the average distance between the atoms of superimposed proteins, were found to be 3.692 Å for the Alphafold model and 1.541 Å for the homology model, respectively, when compared with *Cp*I. The homology model, however, was unable to generate a structure for the C-terminal domain due to the absence of crystal/x-ray structure for modeling. In contrast, Alphafold was able to predict the structure for the C-terminal domain, providing a representation of the [4Fe-4S]$_F$ cluster in the C-terminal domain. The [4Fe-4S] cluster of the H-cluster and the two additional [4Fe-4S] clusters, which are visible in the YASARA-model after alignment with the crystal structure of *Cp*I, are the same clusters that we have incorporated into the AlphaFold model. Given the alignment RMSD of 3 Å between YASARA and AlphaFold, it is reasonable to infer that the positions of the [4Fe-4S] clusters in YASARA closely resemble those in the AlphaFold model. To visually represent the [4Fe-4S] cluster in the C-terminal domain, we extracted a FeS cluster along with its cysteine ligands from the *Cp*I structure. This cluster was then manually integrated into the *Tam*HydS AlphaFold model, binding to the following cysteines in *Tam*HydS: C379, C382, C387, C404. This process was executed using the PyMOL molecular graphics system in "editing" mode. While this approach may not be conventional, it enabled us to visualize the C-terminal [4Fe-4S] clusters in our AlphaFold model. Despite Alphafold's ability to predict protein structures in the absence of crystallographic data, it is important to note that it often struggles with accurately predicting the conformational states, or rotamers, of amino acid side chains as well as the precision of atomic placement.

Figure S2. AGE gels of PCR amplicons.

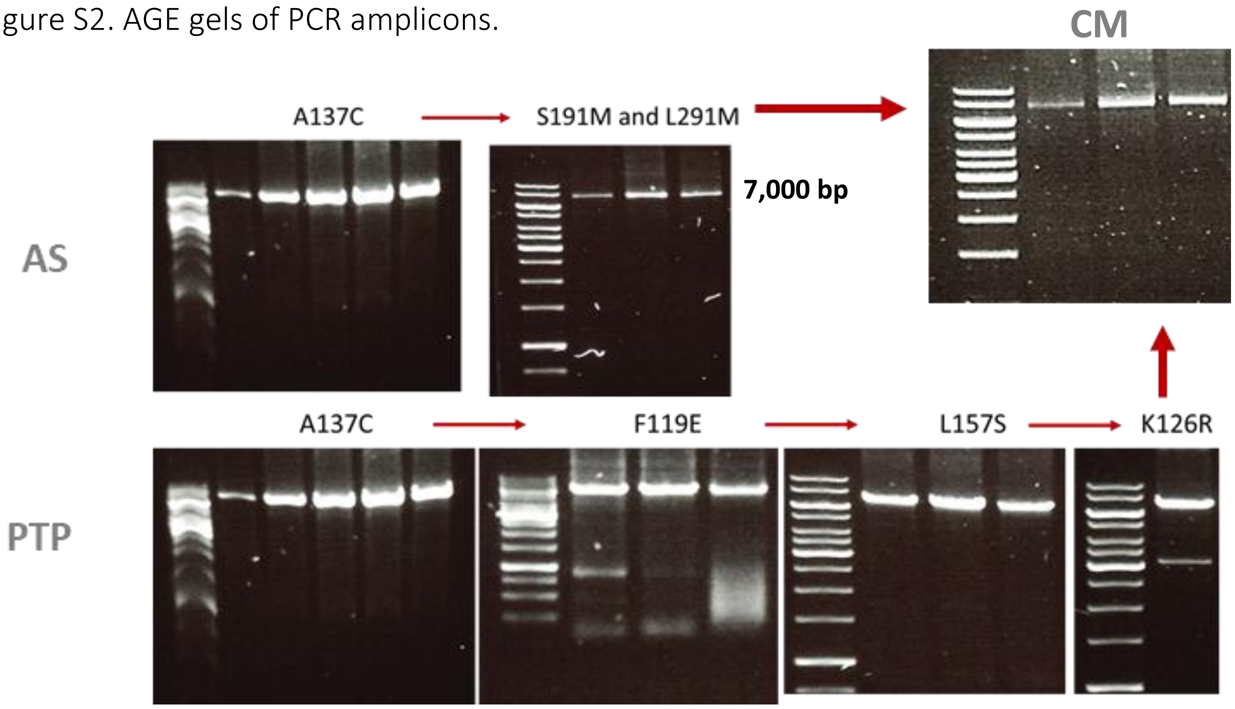

Figure S2. AGE gels of PCR amplicons. A137C, AS, **PTP**, and **CM** were successfully generated using Phusion® High-Fidelity PCR. The arrows indicate the direction of successive point mutations. A band at around 7,000 base pairs indicates the recombinant plasmid with the inserted *Tam*HydS gene (pet-11a+ vector alone has 5,677 bps and the target gene *Tam*HydS has 1,341 bps). Lane 1 (leftmost lane) is loaded with the DNA ladder, while lanes 2 and onwards are the PCR mixtures with varying DMSO concentrations. To produce the **AS** variant from the template plasmid harboring the first A137C mutation, a megaprimer was first generated using the MEGAWHOP method[2] and was subsequently used to create two-point mutations (S191M and L291M).

Figure S3. PAGE gels of expressed and purified variants.

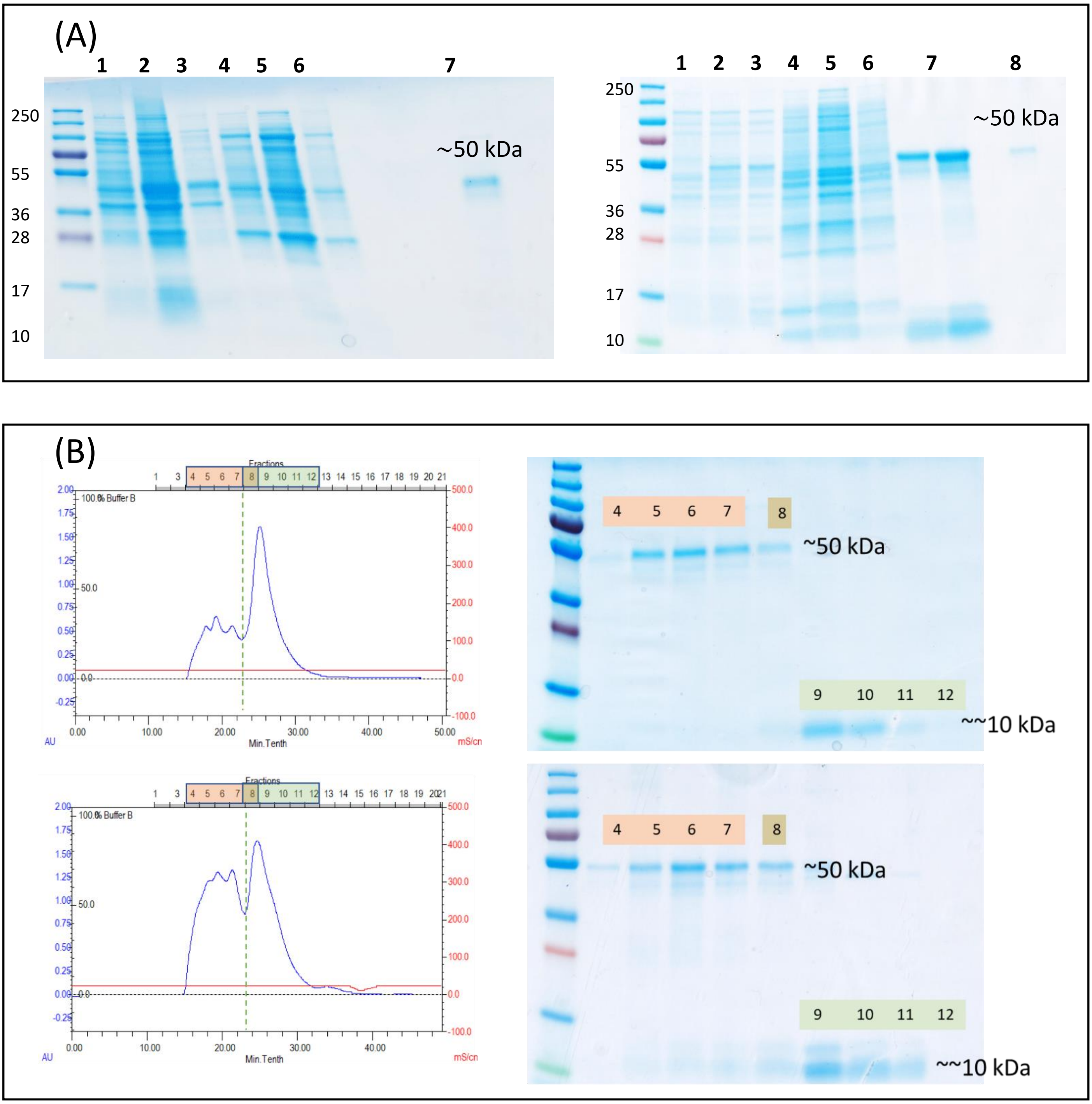

Figure S3. PAGE gels of expressed and purified proteins. Panel A: Lane 1-*E.coli* cells before induction; Lane 2-after induction; Lane 3-lysis pellet; Lane 4-lysis supernatant; Lane 5-Strep-trap column-flow through; Lane 6-binding buffer; Lane 7-StrepTrap-bound protein; Lane 8-Superdex purified protein. All variants were successfully expressed in *E. coli* and displayed decent solubility in aqueous buffers. **AS** was considered pure after Strep-trap purification. **Panel B**: The 10 kDa impurity for **PTP** and **CM** was separated using gel-filtration techniques.

Table S2. Fe:protein ratio before and after reconstitution, as well as after activation with $[2Fe]^{ADT}$.

**Sample**	**Fe:Protein**[a]		
	**Before Reconstitution**	**After Reconstitution**	**After Activation**
**AS**	9.5 ± 0.2	15.6 ± 0.5	18.2 ± 0.2
**PTP**	13.4 ± 0.3	16.0 ± 0.5	18.1 ± 1.2
**CM**	7.1 ± 0.2	15.9 ± 0.1	17.5 ± 2.8
**WT**	13.9 ± 0.9[b]	16.1 ± 0.3[b]	17.8 ± 0.2
**A137C**[c]	8.7	15.8	17.8

[a]Iron quantification data reflects at least two biological replicates, and three technical replicates unless otherwise stated
[b]Data reproduced from reference[11]
[c]Data not collected on biological replicates

Table S2. Fe:protein ratio before and after reconstitution, as well as after activation with $[2Fe]^{ADT}$. Regardless of initial content, all three variants and the **WT** enzyme shared the same final Fe:protein ratio after semi-enzymatic reconstitution. The presence of 16 Fe:protein is in good agreement with the expected four [4Fe-4S] clusters, whereas the incorporation of the diiron cofactor mimic resulted in 18 Fe:protein. The indicated errors are estimated from the Fe assays, and thus, do not reflect uncertainties in protein concentration as determined by Bradford assays with approximately 10% error.

Figure S4. Semi-enzymatic reconstitution of the variants' [4Fe-4S] clusters.

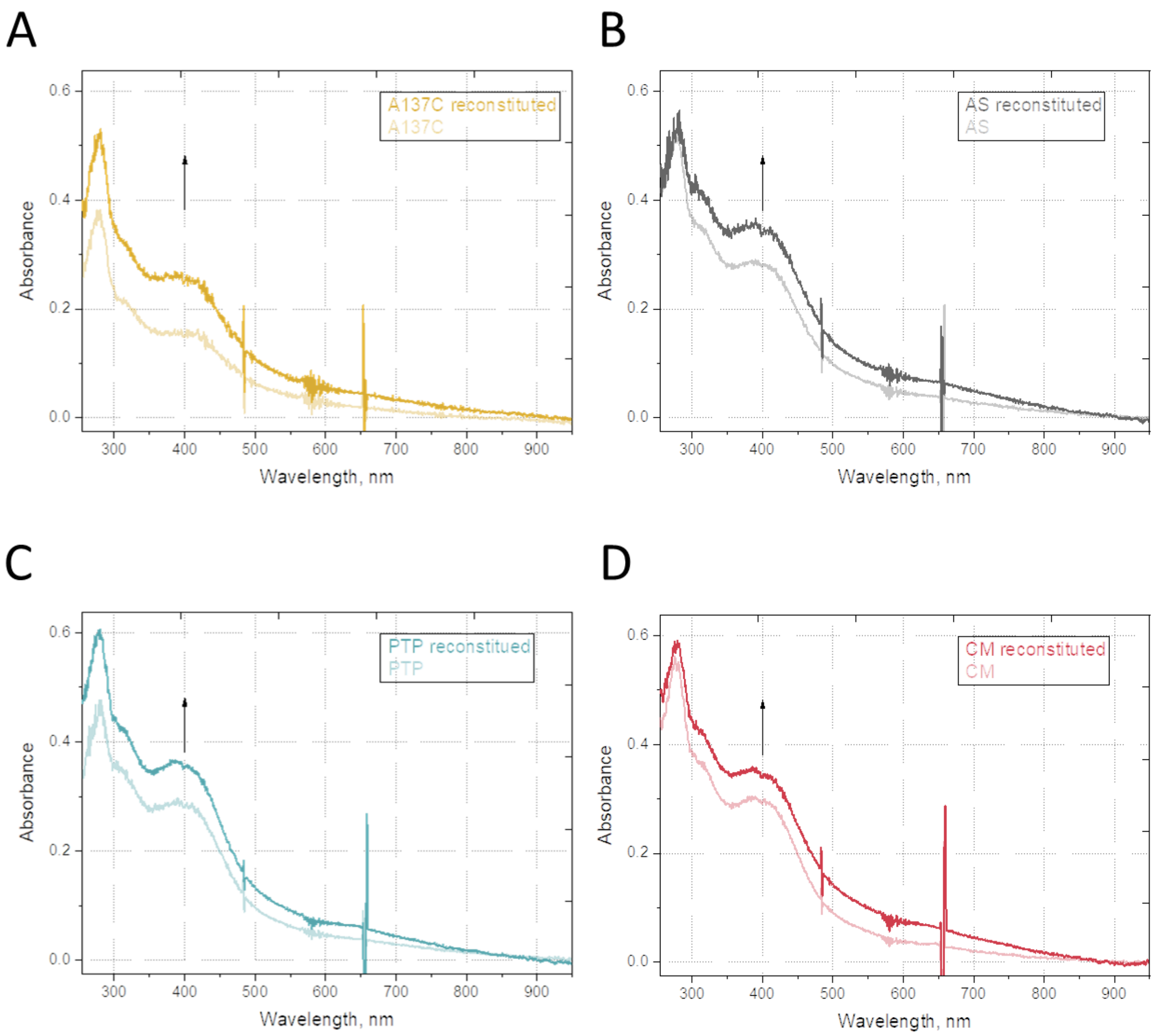

Figure S4. Semi-enzymatic reconstitution of the variants' [4Fe-4S] clusters. An increase of absorbance at 405 nm (arrow) indicates formation of $[4Fe\text{-}4S]^{2+}$ clusters. The UV/Vis spectra were measured in a 1 mm cuvette. The representative UV-Vis spectra of (A) **A137C** (gold), (B) **AS** (gray), (C), **PTP** (cyan), and (D) **CM** (red) depict the progression from the start (light trace, 0 min) to the end (darker trace, 140−150 min) of reconstitution, resulting in the increase of the Fe/protein content (**Table S2**). A solution of 50 ± 5 µM apoprotein in 100 mM Tris-HCl, 150 mM NaCl pH 8.0 was incubated with 500 µM dithiothreitol (DTT) under strictly anaerobic conditions for 10 min at room temperature. The iron and sulfur sources were ferrous ammonium sulfate and L-cysteine, respectively, both added in 1.5-fold molar excess to the desired number of Fe-atoms to be added. Reconstitution was initiated by adding a 1% molar equivalent of 0.5 µM recombinant cysteine desulfurase (*E. coli* IscS).

Figure S5. EPR spectra of visualizing the [4Fe-4S]$^{+}$ clusters of NaDT-reduced apo-*Tam*HydS WT and variants.

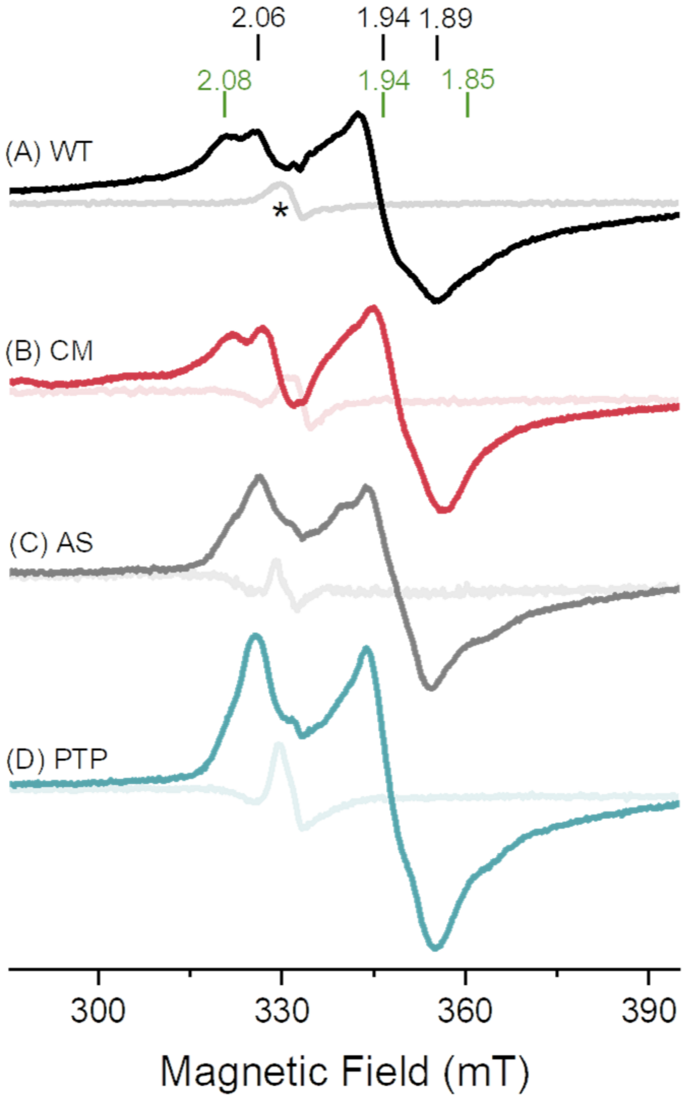

	[4Fe-4S]$^{+}$ wide			[4Fe-4S]$^{+}$ narrow		
**WT**	2.08	1.94	1.85	2.06	1.94	1.89
**CM**	2.09	1.94	1.85	2.06	1.94	1.89
**AS**	2.08	1.93	1.85	2.06	1.93	1.89
**PTP**	2.08	1.93	1.85	2.06	1.93	1.89

Figure S5. EPR spectra of visualizing the [4Fe-4S]$^{+}$ clusters of NaDT-reduced apo-*Tam*HydS WT and variants. (A) X-band EPR spectra of reconstituted apo-*Tam*HydS **WT**, data from Land et al., 2020[11]. Gray: as-prepared; black: sodium dithionite (NaDT) reduced. (B) reconstituted apo-**CM**: light red: as prepared; dark red: NaDT-reduced. (C) reconstituted apo-**AS**: light gray: as prepared; dark gray: NaDT-reduced (D) reconstituted apo-**PTP**: light cyan: as prepared; dark cyan: NaDT-reduced. The g-values in green (wide) and black (narrow) were previously determined to be two contributing components typical for [4Fe–4S]$^{+}$ clusters. The weak signal marked with * appearing at g = 2.02 is attributed to a trace amount of [3Fe–4S]$^{+}$. EPR settings for **WT**: T=17 K; modulation frequency 100 kHz, amplitude 15 G; microwave frequency 9.4 GHz, power 8 mW. EPR settings for the variants: T=10 K; modulation frequency 100 kHz, amplitude 15 G; microwave frequency 9.4 GHz, power 80 µW. The table on the right tabulates the g-values for the wide and narrow components of the [4Fe–4S]$^{+}$ clusters.

Table S3. Tabulated g-values of selected states reported for the H-cluster.

Group & Subclass	[FeFe] hydrogenase	Species	g-values			Reference
			g_1	g_2	g_3	
**A-M1**	***Cr*HydA1**	H_{ox}	2.100-2.101	2.037-2.039	1.996-1.998	13-15
		H_{ox}-CO	2.052	2.007	2.007	15
		$H_{sred}H^+$	2.145	1.860	1.860	16
		H_{hyd}	2.069-2.080	1.935-1.938	1.870-1.880	13, 16-18
		$H_{hyd}H^+$	2.073	1.935	1.881	19
**A-M2(D)**	***Dd*H**	H_{ox}	2.100	2.040	1.998	20
		H_{ox}-CO	2.065	2.007	2.001	20
		H_{trans}	2.060	1.960	1.890	20
**A-M3**	***Cp*I**	H_{ox}	2.097	2.039	1.999	21
		H_{ox}-CO	2.072	2.006	2.006	21
**C-M2f**	***Tm*HydS**	H_{ox}	2.113	2.045	2.001	22
		H_{ox}-CO	2.047	2.018	2.007	22
**D-M2e**	***Tam*HydS**	H_{ox}-R1	2.109	2.053	2.010	11, 23
		H_{ox}-R2	2.099	2.044	2.010	11, 23
		H_{ox}-CO	2.034	2.023	2.023	23
		State 2	2.041	2.022	2.022	11
		State 2*	2.046	2.023	2.020	23
	***Tam*HydS-AS variant**	H_{ox}-R2	2.109	2.048	2.011	This work
		H_{ox}-CO	2.030	2.022	2.017	This work
	***Tam*HydS-PTP variant**	H_{ox}-R1	2.100	2.050	2.012	This work
		H_{ox}-R2	2.109	2.034	2.008	This work
		H_{ox}-CO	2.038	2.023	2.018	This work
	***Tam*HydS-CM variant**	H_{ox}-R1	2.101	2.051	2.005	This work
		H_{ox}-R2	2.109	2.035	2.009	This work
		H_{ox}-CO	2.038	2.023	2.017	This work
		State 2	2.050	2.022	2.019	This work

The asterisk (*) indicates g-values from the simulated spectrum of *Tam*HydS variant, **E252V**, in Cabotaje et al., 2023.[23] **State 2** in *Tam*HydS **WT** was reported as State A2 in Land et al., 2020 ($g_{\parallel}$ =2.041 and $g_{\perp}$ = 2.022), with the State A2 corresponding to **State 2** of the *Tam*HydS variants reported in Cabotaje, et al., 2023 ($g_{\parallel}$ = 2.046, $g_{\perp}$ = 2.0225, 2.0196, average 2.021).[11, 23]

Table S3. Tabulated g-values of selected states reported for the H-cluster in Group A *Cr*HydA1, *Dd*H and *Cp*I, Group C *Tm*HydS, and Group D *Tam*HydS. The g-values of the *Tam*HydS variants, **AS**, **PTP**, and **CM**, studied in this work are also included.

Figure S6. Temperature and power dependence studies of H_{ox}-CO signals

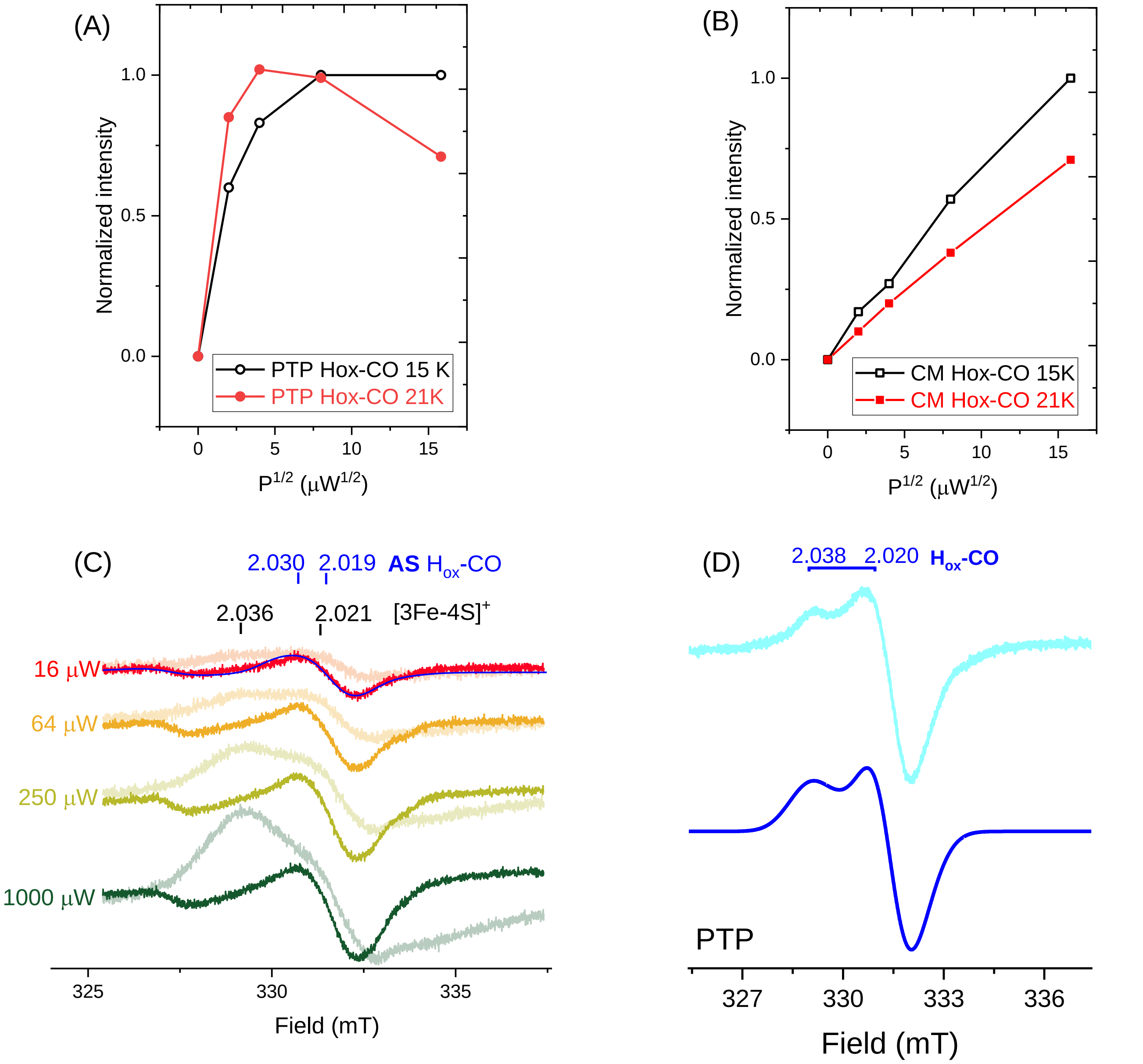

Figure S6. Dependence of the **(A) PTP** H_{ox}-CO and **(B) CM** H_{ox}-CO signal intensity on the square root of microwave power at 15 K (black) and 21 K (red). Despite sharing the same set of g-values for H_{ox}-CO, **PTP** and **CM** exhibit distinct relaxation properties. **(C)** Dependence of the **AS** H_{ox}-CO signal intensity (dark colors) on the microwave power versus the signal for $[3Fe\text{-}4S]^+$ cluster (light colors) at 21 K. The simulated spectrum for **AS** H_{ox}-CO signal from **Fig. 2A** is shown in blue. The $[3Fe\text{-}4S]^+$ signal from apo-**PTP** was used as a reference (refer to **Fig. S5**), with the g-values equivalent to those of the apo-**AS** sample; however, apo-**PTP** has a stronger $[3Fe\text{-}4S]^+$ signal, making it a better reference for comparison. The H_{ox}-CO and $[3Fe\text{-}4S]^+$ signals exhibited contrasting features: (a) the former displayed a narrower shape compared to the latter, and (b) their relaxation properties differed when examining signal intensity at varying powers. EPR settings: Modulation frequency 100 kHz, amplitude 10 G; Microwave frequency 9.4 GHz; power 16 µW (red), 64 µW (orange), 250 µW (light green), and 1000 µW (dark green). **(D)** Experimental (cyan) and simulated (blue) spectra for the H_{ox}-CO signal of CO-flushed **PTP** at T = 21K; modulation frequency = 100 kHz; amplitude = 10 G; microwave frequency = 9.4 GHz; microwave power = 64 µW.

Figure S7. EPR spectra of H_2-reduced holo-*Tam*HydS WT and variants.

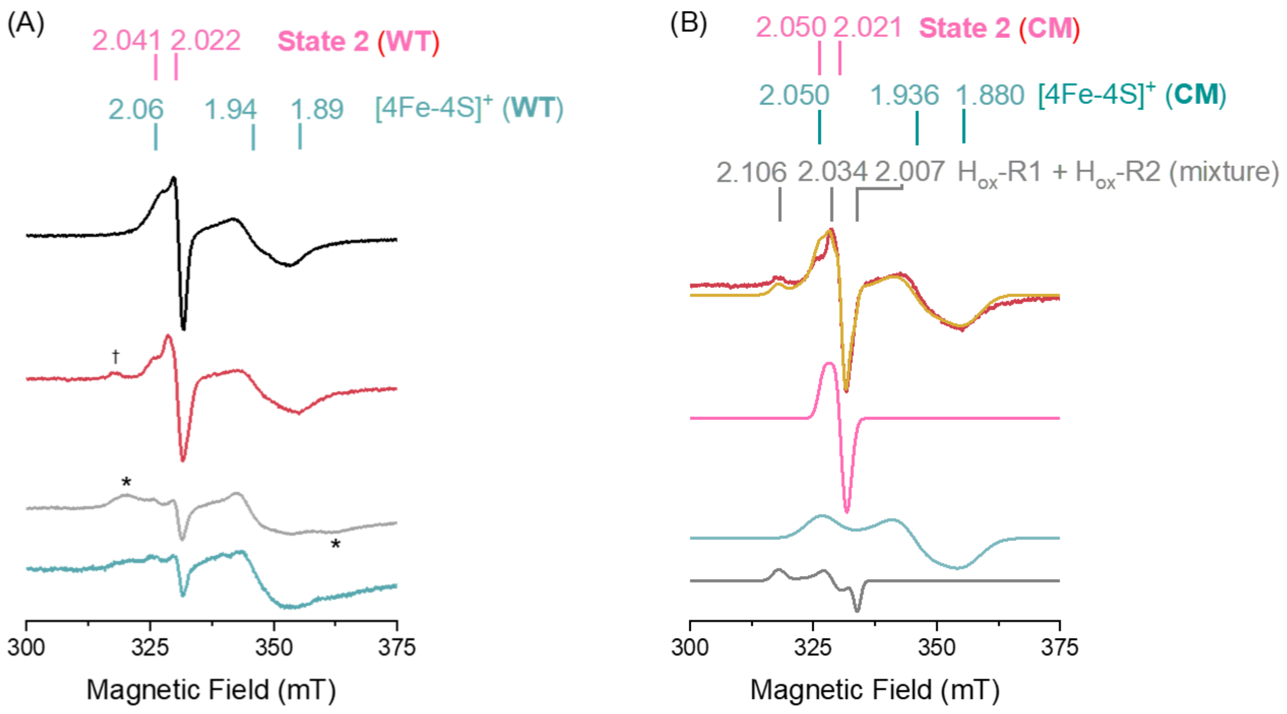

Figure S7. (A) EPR spectra H_2-reduced holo-*Tam*HydS WT and variants. Reduction of holo-*Tam*HydS **WT** (black trace) and variants, **CM** (red trace), **AS** (gray trace), and **PTP** (cyan trace) by H_2, resulting in the formation of **State 2** (pseudo-axial signal) with parallel accumulation of reduced $[4Fe–4S]^+$ (rhombic signal). EPR settings: T=10 K; Modulation frequency 100 kHz, amplitude 10 G; Microwave frequency 9.4 GHz; power 80 µW. The **WT** spectrum was obtained from Land et al., 2020 with contributions mainly from the "narrow" rhombic $[4Fe–4S]^+$ component having $g_{1,2,3}$ = 2.06, 1.94, 1.89 (not simulated, cyan bars in panel A) and from **State 2** having $g_{\parallel}$ = 2.041, $g_{\perp}$ = 2.022 (simulated, magenta bars in panel A).[14] The dagger (†) in **CM** (red trace) indicates small contributions from a mixture of H_{ox}-R1 and H_{ox}-R2. The asterisks (*) in **AS** (gray trace) correspond to the trace signal from the "wide" rhombic component of $[4Fe-4S]^+$ (**refer to Fig. S5**), which is presumed to arise from a population of apo-**AS**. This assignment is based on our previous data from Land et al., 2020.[14] **(B)** The experimental spectrum of **CM** (red trace) overlaid with its simulated spectra (gold trace). The three individual components contributing to the overall simulated spectrum for **CM** are shown in magenta for **State 2** ($g_{1,2,3}$ = 2.050, 2.022, 2.019; pseudo-axial $g_{\parallel}$ = 2.050, $g_{\perp}$ = 2.021), cyan for the "narrow" $[4Fe–4S]^+$ component ($g_{1,2,3}$ = 2.050, 1.936, 1.880), the gray trace reflects the minor contributions from a mixture of H_{ox}-R1 and H_{ox}-R2 that was treated as a single component. **State 2** was reported to have $g_{\parallel}$ = 2.046, $g_{\perp}$ = 2.0225, 2.0196 (average 2.021) after simulation of a spectrum from a *Tam*HydS variant, **E252V**.[11, 23] The $g_{average}$ for **State 2** in **WT**, **E252V**, and in **CM** are 2.032, 2.029, and 2.030, respectively; and for the narrow $[4Fe-4S]^+$ component in **WT =** 1.963 and in **CM** = 1.955. The $g_{anisotropy}$ for **State 2** in **WT**, **E252V,** and in **CM** is 0.019, 0.026, and 0.031, respectively; and for the "narrow" $[4Fe-4S]^+$ component 0.170 in both **WT** and **CM**. The highly similar $g_{average}$ and $g_{anisotropy}$ for **State 2** suggest minor changes in the g-tensor rhombicity of the H-cluster, indicating minor shifts in its electronic structure. The highly similar $g_{average}$ and $g_{anisotropy}$ for $[4Fe-4S]^+$ between the **WT** and **CM** samples suggest that the electron-transfer relays remain unaltered.

Supporting Note 1

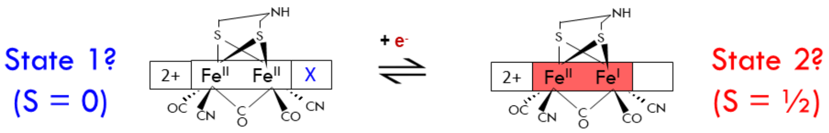

State 1 and **State 2** refer to two still only partially characterized H-cluster states. **State 1** has to-date only been observed in variants of *Tam*HydS in which the proton transfer pathway has been altered, either through introduction of the Group A PTP (this report), or via disruption of the proposed native proton transfer pathway.[23] This state was previously described to be an “over-oxidized” di-ferrous species with an electronic configuration analogous to the terminal hydride state H_{hyd} or the sulfide inhibited state H_{inact} (*i.e.* $[4Fe\text{-}4S]^{2+}\text{-}[Fe^{II}Fe^{II}]$) observed in Group A [FeFe] hydrogenases; however, **State 1** is proposed to be ligated by an aqua (or hydroxide) ligand instead.[23] The preference for aqua (or hydroxido) ligation in these variants has been proposed to arise from a shift in the positioning of the ADT-amine due to a loss of hydrogen bonding upon modification of the proton transfer pathway.[23] Sulfide binding is unlikely to be involved in the formation of **State 1**, as it is generated in the absence of sulfide sources or O_2, *e.g.*, with N_2 or CO (as exemplified by the PTP-variant, **Fig. 3C**). We note that **State 1** has previously been observed also in loss-of-function *Tam*HydS variants that lack any sulphur containing amino acid in their active-site pocket, further arguing against thiol binding.[23]

Structural support for the notion of water ligation to the H-cluster has been reported. High resolution X-ray crystallographic studies of the [FeFe] hydrogenase derived from *Cp*I have elucidated that the experimental value for the distance between the oxygen atom of a water molecule (referred to as the “distal water”) and the distal iron is 2.38 Å.[24] The bond distance for Fe(II)-O for aqua ligands typically ranges from 2.095 Å to 2.13 Å. Despite the distal water oxygen-distal iron distance being slightly longer than the anticipated Fe(II)-O distance, the bond length of Fe-O can be influenced by secondary coordination sphere effects, a concept illustrated in a study by Mukherjee et al. in 2008, where it was shown that the bond length in Fe(III)-OH complexes can be elongated by the presence of intramolecular hydrogen bonds.[25] Furthermore, Budria et al. in 2006 demonstrated that monomeric Fe(III) model complexes, which lack hydrogen bonds, exhibit shorter Fe-O bond lengths.[26]

Meanwhile, **State 2** was proposed to be a relatively more reduced species with a tentatively assigned oxidation state similar to H_{ox} (*i.e.* $[4Fe\text{-}4S]^{2+}\text{-}[Fe^{I}Fe^{II}]$), due to the red-shift observed in FTIR spectroscopy relative to **State 1** and the (pseudo-)axial EPR signal associated with this state. However, the H_{ox} state commonly displays a clear rhombic EPR signal, and the more axial nature of **State 2** implies a difference in electronic distribution.[27] **State 2** has been observed in **WT** as well as several variants, upon H_2 treatment. [11, 23]

State 1 would represent an inhibited state according to the above reasoning, providing some measure of protection against irreversible O_2-induced H-cluster degradation. **State 2** is potentially catalytically relevant, and could represent a form of the H-cluster residing in an H_{ox}-like oxidation state coupled to a reduced F-cluster, as it only appears under reducing conditions. However, this remains to be verified.

The FTIR spectra of the **PTP** variant is dominated by **State 2** under an H_2 atmosphere. The single-point variant A137C also exhibits a clearly discernible population of **State 2**, although extended H_2 treatment only appears to increase the H_{red} exclusively at the expense of H_{ox} (**Fig. 3A**). The origin of this difference in H-cluster state accumulation has not been identified. However, considering that **PTP** displays similar H_2 formation rates as **WT**, it suggests that **State 2** is catalytically competent.

Figure S8. Spectral characterization

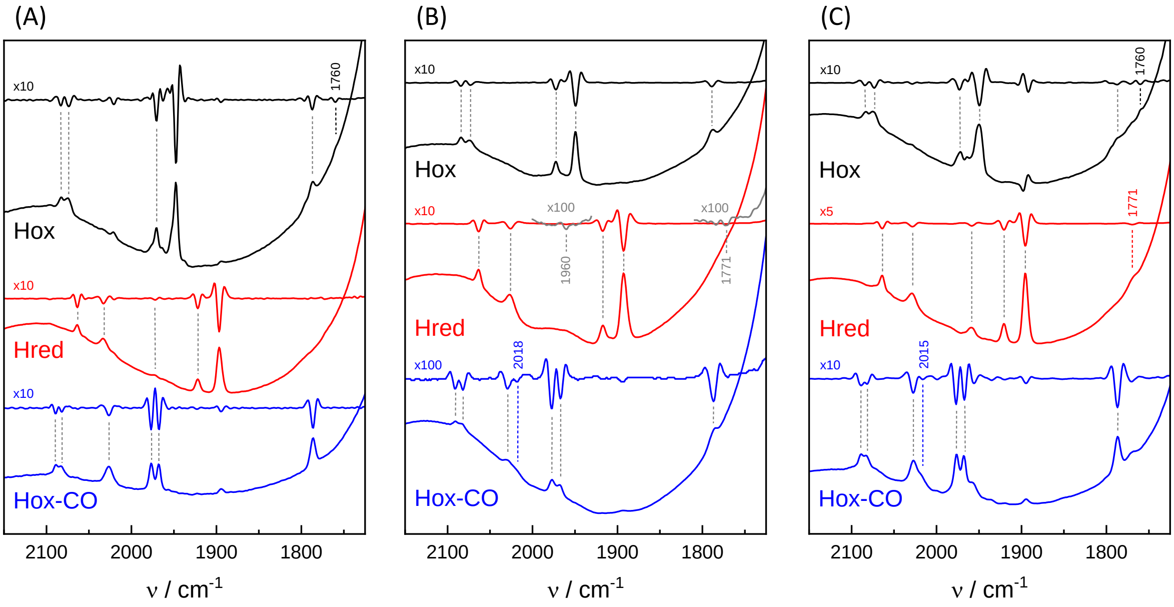

Figure S8. Spectral characterization. Identification of the CO/CN band patterns of different H-cluster states in *Tam*HydS **WT (A)**, **AS (B)**, and **CM (C)**. Pure spectra were calculated by subtraction of absorbance spectra at different time points from H_2/N_2 or CO/N_2 titrations (see **Figs. S9-10**). The second derivative of such spectra allows identifying the individual frequencies without manual baseline correction. Legend: H_{ox} (N_2, black), H_{red} (H_2, red), H_{ox}-CO (CO, blue). Further observations: The second derivative of the H_{red} spectrum includes weak spectral contributions at 1960 cm^{-1} *and* 1771 cm^{-1} (100x magnified, grey, in panel **(B)**), hinting at the presence of μH- *and* μCO-derivates of H_{red}. The H_{ox}-CO state shows a red-shifted shoulder near the highest CO band (2028/2018 cm^{-1} in *Tam*HydS **AS** and 2026/2015 cm^{-1} in *Tam*HydS **CM**). In the oxidized form, the *Tam*HydS **WT** and the **CM** variant adopt a mixture of H_{ox} and H_{ox}H (note the inhomogeneity of the spectra). Here, the low-frequency band at 1760 cm^{-1} cannot be assigned to a known H-cluster state.

Table S4. Summary of FTIR bands vs Group A.

Comparison of H-cluster states H_{ox}, H_{red}, and H_{ox}-CO as observed in *Tam*HydS **WT**, **AS**, and **CM** to the signature of Group A or "standard" [FeFe] hydrogenases *Dd*H, *Cr*HydA1, and *Cp*I. Notable differences include the ~14 cm^{-1} downshift of the µCO band in H_{ox}, the ~13 cm^{-1} upshift of the coupled vCO_{asym} in H_{ox}-CO, and the ~22 cm^{-1} downshift of the µCO band in H_{ox}-CO.

	***Dd*H**	***Cr*HydA1**	***Cp*I**	***mean***	***Tam*HydS**			***Tam*HydS - *mean***		
					**WT**	**AS**	**CM**	**ΔWT**	**ΔAS**	**ΔCM**
**Hox**	2093	2088	2082	2088	2083	2084	2082	-5	-4	-6
	2079	2070	2070	2073	2073	2072	2073	0	-1	0
	1965	1964	1970	1966	1971	1972	1972	5	6	6
	1940	1940	1947	1942	1947	1949	1949	5	7	7
	1802	1802	1802	1802	1788	1789	1788	-14	-13	-14
**Hred**	2079	2070	2063	2071	2064	2063	2063	-7	-8	-8
	2041	2033	2040	2038	2032	2026	2027	-6	-12	-11
	1965	1961	1961	1962	1962	1960	1960	0	-2	-2
	1916	1915	1916	1916	1922	1917	1921	6	1	5
	1894	1891	1900	1895	1896	1892	1896	1	-3	1
**Hox-CO**	2096	2091	2090	2092	2088	2090	2088	-4	-2	-4
	2088	2081	2076	2082	2082	2080	2080	0	-2	-2
	2016	2012	2015	2014	2026	2028	2026	12	14	12
	1971	1968	1973	1971	1978	1978	1976	7	7	5
	1963	1962	1968	1964	1966	1967	1966	2	3	2
	1810	1808	1806	1841	1786	1785	1786	-22	-23	-22

Figure S9. Kinetics of state conversions.

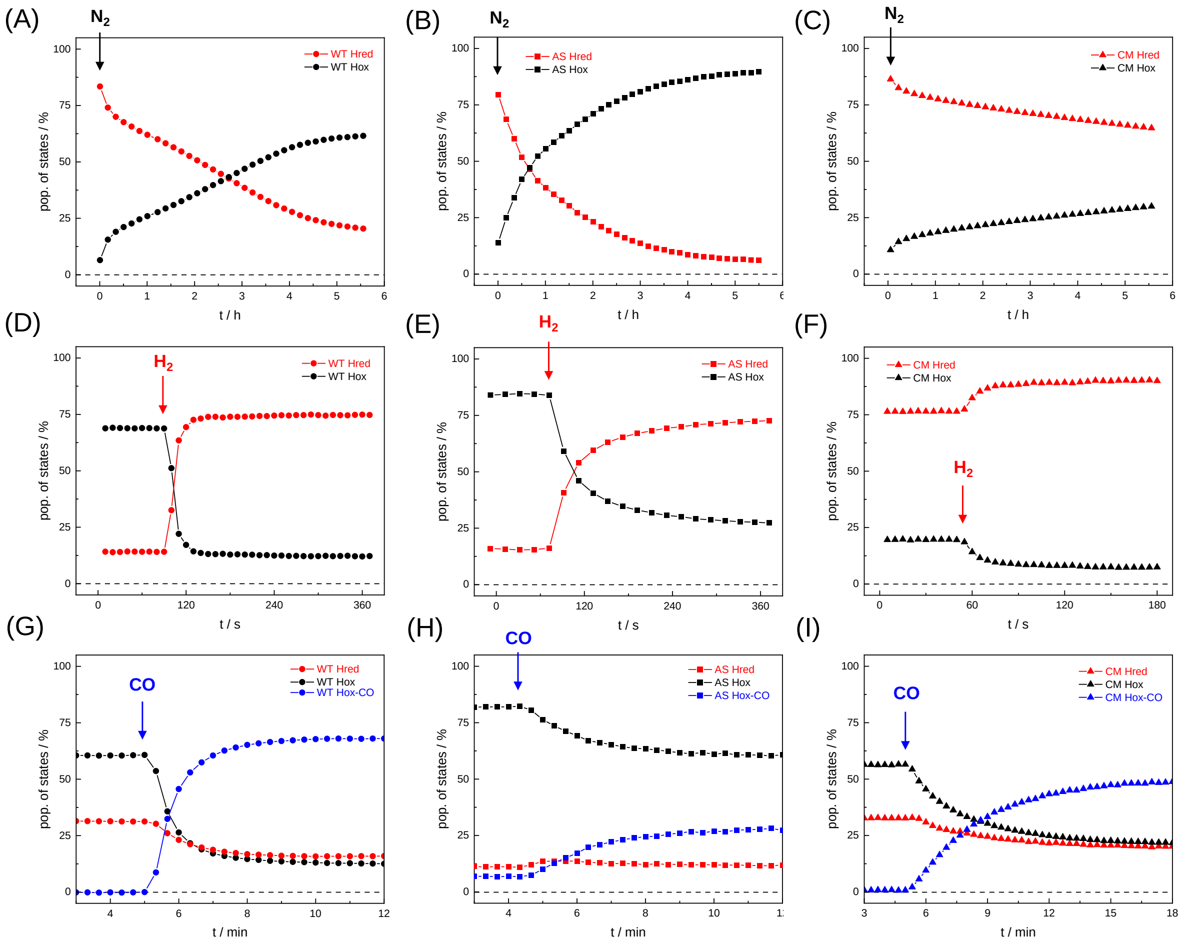

Figure S9. Kinetics of state conversions. Upper row: In the presence of N_2, the oxidized state H_{ox} accumulates over the one-electron reduced state H_{red}. This process of auto-oxidation (reducing equivalents are released as H_2 due to the catalytic activity of the enzymes) is slow in **WT (A)** and **AS (B)** and very slow and incomplete in **CM (C)**. **Middle Row**: In the presence of 1% H_2, the one-electron reduced state H_{red} accumulates over H_{ox}. H_2-induced reduction of the H-cluster is very fast in **WT (D)**, and moderately fast in **AS (E)** and **CM (F)**. In all samples, H_2 oxidation is significantly faster (tens of seconds) than the auto-oxidation process (~hours). Lower row: In the presence of 99% CO, H_{ox}-CO accumulates over H_{ox} and H_{red}. Compared to standard [FeFe] hydrogenase, CO inhibition is slow and incomplete but fastest in **WT (G)** and slower in **AS (H)** and **CM (I)**. In all samples, CO reacts with the oxidized H-cluster (H_{ox}) to a greater extent than with the reduced H-cluster (H_{red}).

Figure S10. Reaction with H_2 and CO monitored in FTIR.

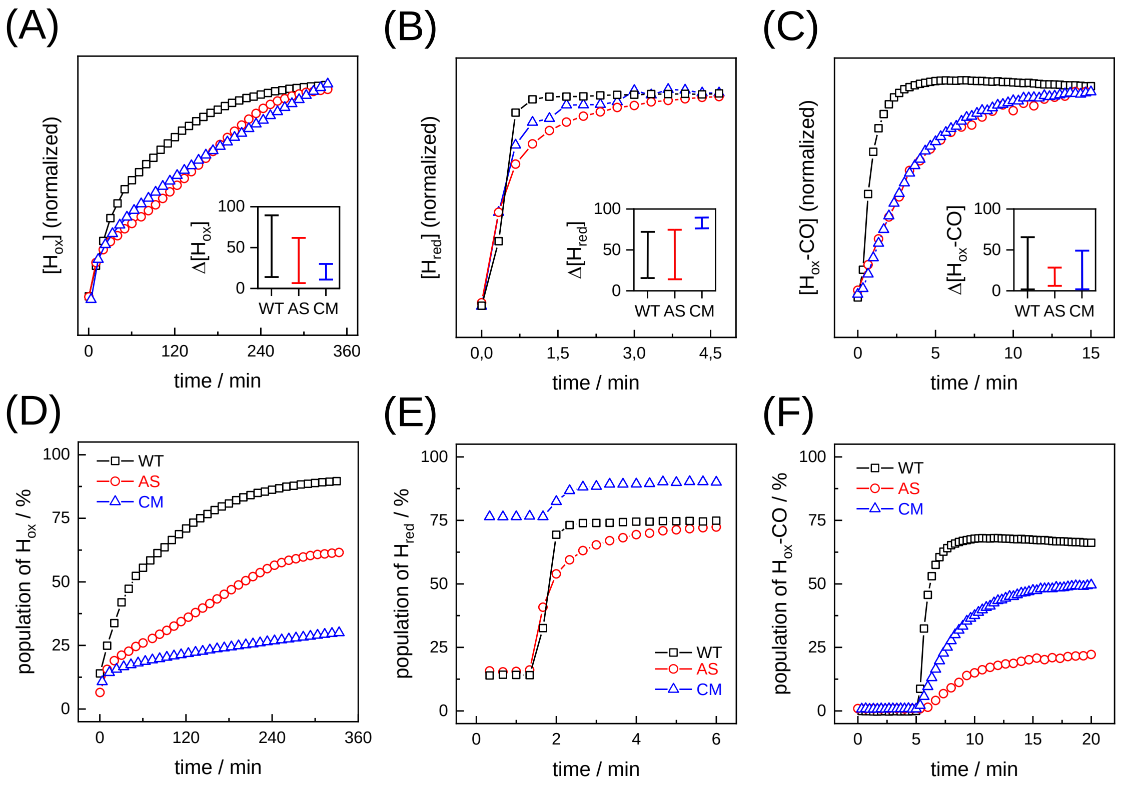

Figure S10. Normalized kinetics of state conversions. (A) In the presence of N_2, the oxidized state H_{ox} accumulates in *Tam*HydS **WT** (black) and variants **AS** (red) and **CM** (blue). The rate of auto-oxidation is slow for all samples (WT > AS ≈ CM). The inset illustrates that the increase of the H_{ox} population over the course of the experiment is semi-quantitative ($\Delta[H_{ox}]$ > 50%) in **WT** or **AS** and only about 20% in **CM**. **(B)** In the presence of 1% H_2, the one-electron reduced state H_{red} accumulates in *Tam*HydS **WT** (black) and variants **AS** (red) and **CM** (blue). The rate of reduction is fast in all samples (WT > CM > AS). Inset: the conversion is semi-quantitative ($\Delta[H_{red}]$ > 50%) in **WT** or **AS** and only about 10% in **CM**. Compared to the inset in panel **(A)**, it becomes clear that the **CM** variant is very difficult to oxidize and always shows high levels of H_{red}, which very likely contributes to the poor conversion efficiency under H_2. **(C)** In the presence of 99% CO, the inhibited state H_{ox}-CO accumulates in **WT** (black) and variants **AS** (red) and **CM** (blue). CO inhibition is slow for all samples (**WT** >> **AS** ≈ **CM**). In variance to oxidation (panel **(A)**) or reduction (panel **(B)**), the inset shows that the conversion is semi-quantitative ($\Delta[H_{ox}$-CO] >50%) in **WT** or **CM** and only about 20% in **AS**. Panels **D–F** show the same data as in panels **A–C** without normalization. All data taken from **Fig. S8**.

Figure S11. Second scan CV traces of *Tam*HydS and variants.

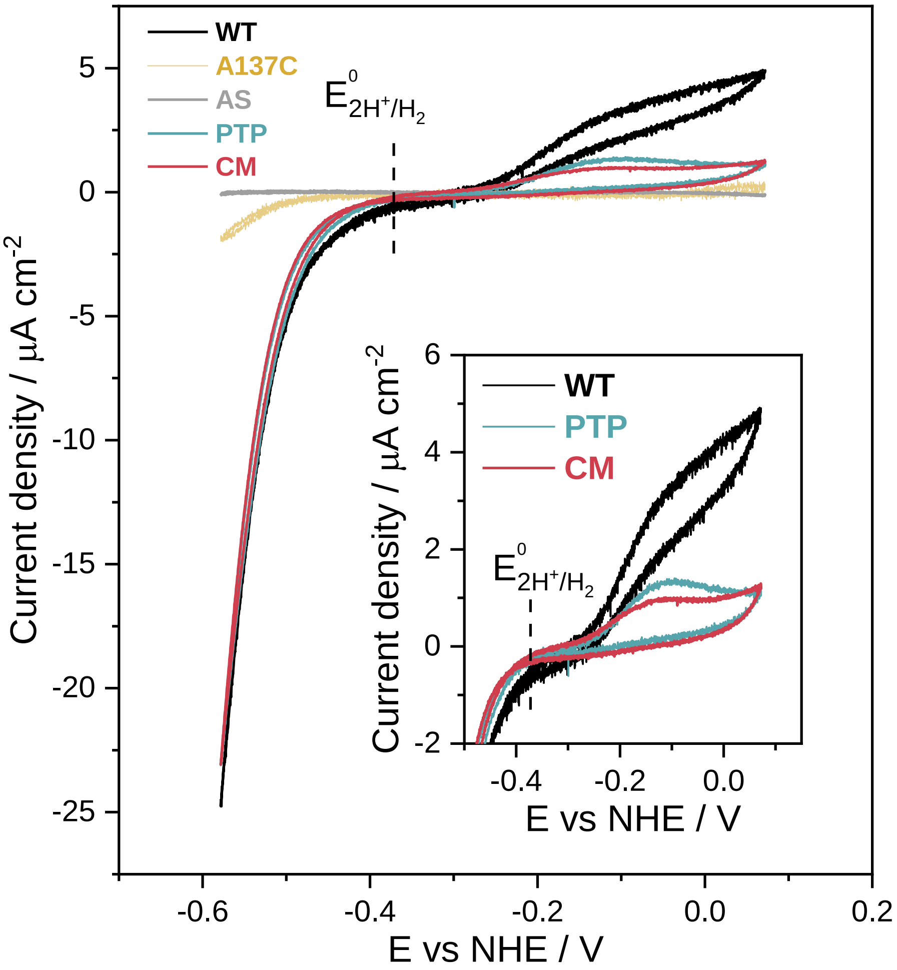

Figure S11. Second scan CV traces of *Tam*HydS and variants. Shown are representative CV traces (second scan) observed for **WT** (black), **A137C** (gold), **AS** (gray), **PTP** (cyan), and **CM** (red) subtracted with the blank electrode (no immobilized enzyme). Inset: Zoom in of the regions close to the formal reduction potential of the $2H^+/H_2$ couple. Experiments were performed at pH 6.0, 40°C, 1 atm H_2, scan rate 2 mV/s, 3 krpm, following immobilization of the enzyme on a PGE electrode; all potentials given in V vs. NHE.

Figure S12. Changes in current densities with scan number.

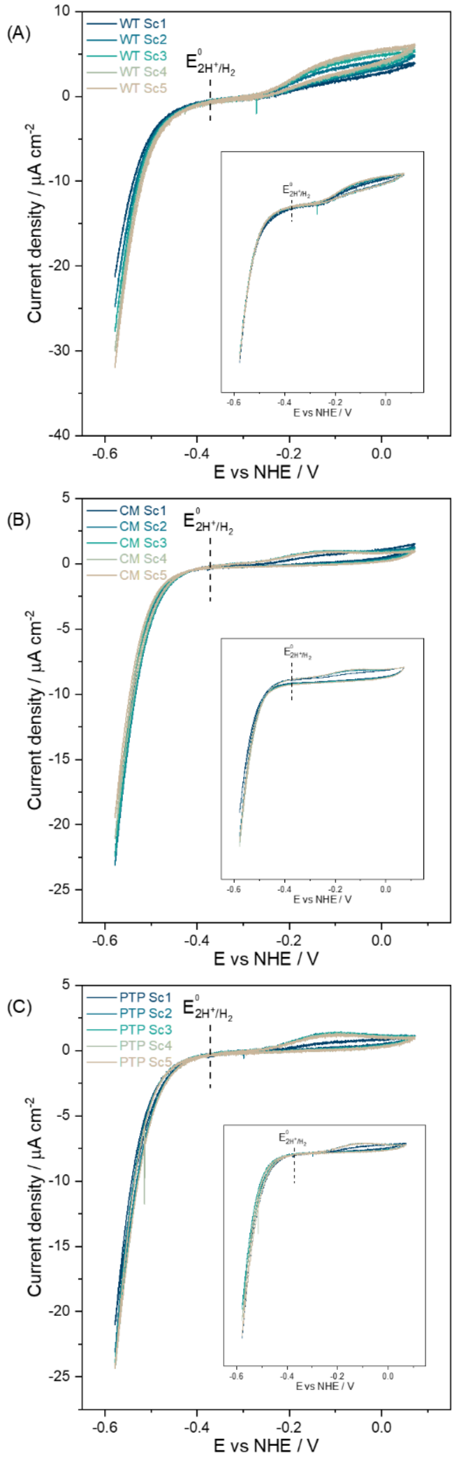

Figure S12. Changes in current densities with scan number. Five successive CV traces by **(A) WT**, **(B) CM**, and **(C) PTP**. Experiments were performed at pH 6.0, 40°C, 1 atm H_2, scan rate 2 mV/s, 3 krpm, following immobilization of the enzyme on a PGE electrode; all potentials given in V vs. NHE. Inset: Normalized CV scan traces 1-4 to the highest oxidation current value of the 5th scan.

Table S5. Tabulated specific activities

	H_2 oxidation		H_2 evolution
	**BV, –0.359 V[6]**	**MB, 0.008 V[6]**	**MV, –0.446 V[6]**
**WT**	1.95 ± 0.27	22.1 ± 0.8	0.25 ± 0.03
**AS**	0.48 ± 0.05 *(*)*	0.38 ± 0.03 *(*)*	Not detected
**PTP**	2.43 ± 0.52 *(ns)*	3.70 ± 0.29 *(*)*	0.60 ± 0.01 (*ns*)
**CM**	1.67 ± 0.03 *(ns)*	2.88 + 0.89 *(*)*	36.2 ± 1.8 *(*)*

Table S5. Tabulated specific activities of *Tam*HydS **WT** and variants in U/mg where one unit (U) of activity catalyzes 1 µmol of H_2 oxidized or produced per min under the utilized assay conditions, which are described in the experimental procedure section. The redox potentials of the mediators—Benzyl Viologen (BV^{2+} →$BV^{\bullet+}$), Methylene Blue (MB^{+} to Leucomethylene Blue, LMB), Methyl Viologen ($MV^{\bullet+}$ →MV^{2+})—are given in V, E°' vs. SHE[6]. **AS** had negligible H_2-evolution activities that were below the detection limit of the GC equipment. Results from the one-way ANOVA (Tukey's HSD Test for multiple comparisons) are indicated as: statistically significantly different at the p = 0.05 level *(*)* and not statistically significantly different *(ns)* based on mean exam scores between **WT** enzyme and variants. Data are presented as a mean of 2 biological replicates, each with 2-3 technical replicates with error values showing SEM.

Figure S13. Chronoamperometry measurements

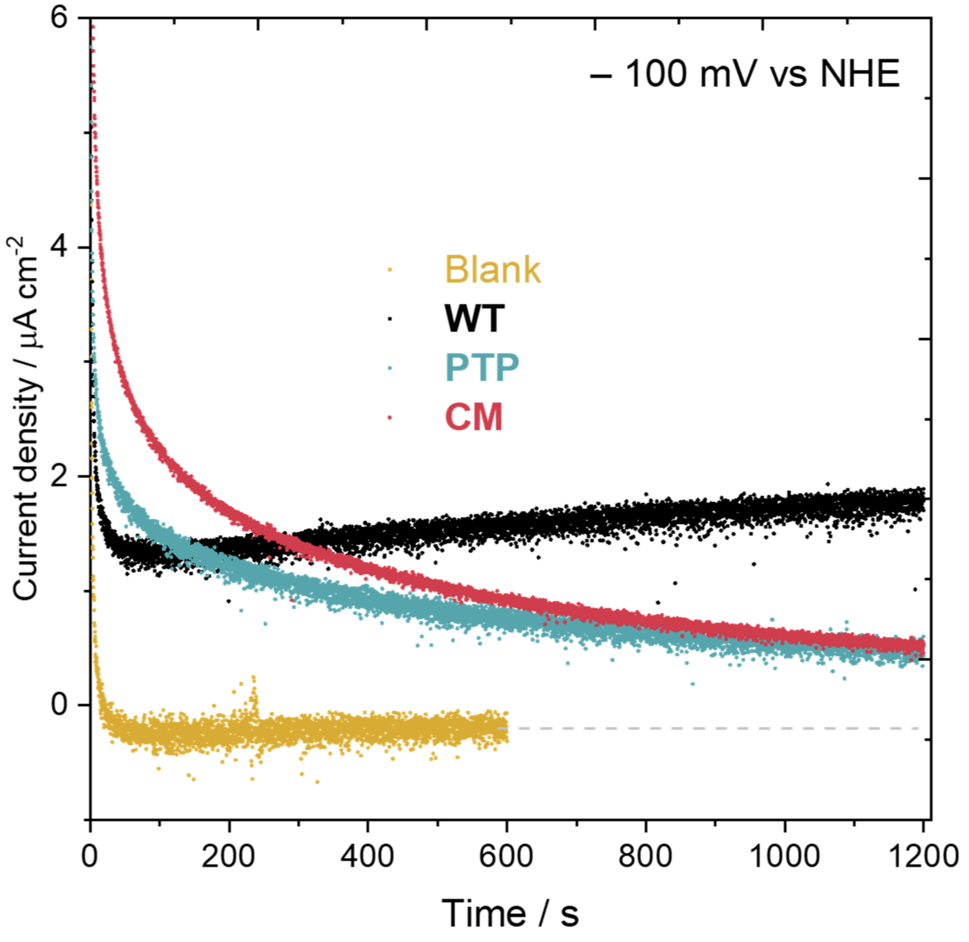

Figure S13. Chronoamperometry measurements of *Tam*HydS **WT** (black) and variants, **CM** (red), and **PTP** (cyan) immobilized in deaerated PGE working electrodes recorded at – 100 mV vs NHE, pH 6.0, 40°C, 1 atm H_2, and rotation = 3 krpm. The signal of the blank (gold) is from the bare electrode without immobilized enzyme. Sustained electrolysis at –100 mV vs NHE caused a slow but distinct decrease in oxidative current on a minute time-scale for both the **CM** and **PTP** variants, further confirming their inactivation during H_2 oxidation catalysis.

Figure S14. A. Cyclic voltammograms of AS and A137C

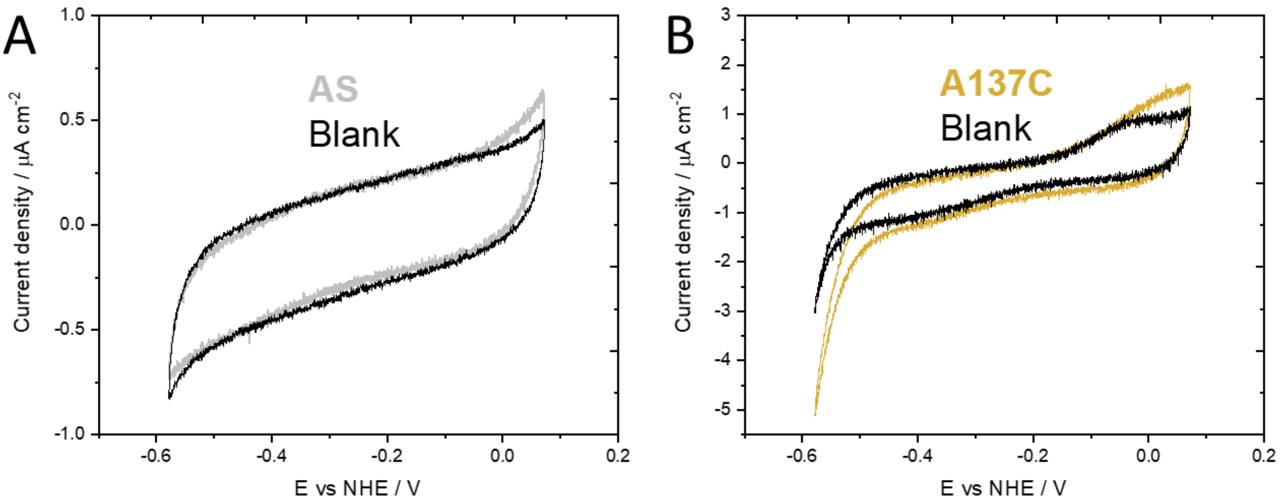

Figure S14. Representative CV traces of *Tam*HydS variants (A) AS (gray) and **(B) A137C** (gold) overlaid with the blank (black). Measurements recorded at pH 6.0, 40°C, 1 atm H_2, scan rate 2 mV/s, and rotation = 3 krpm.

Figure S15. DET vs MET electrochemistry experiments

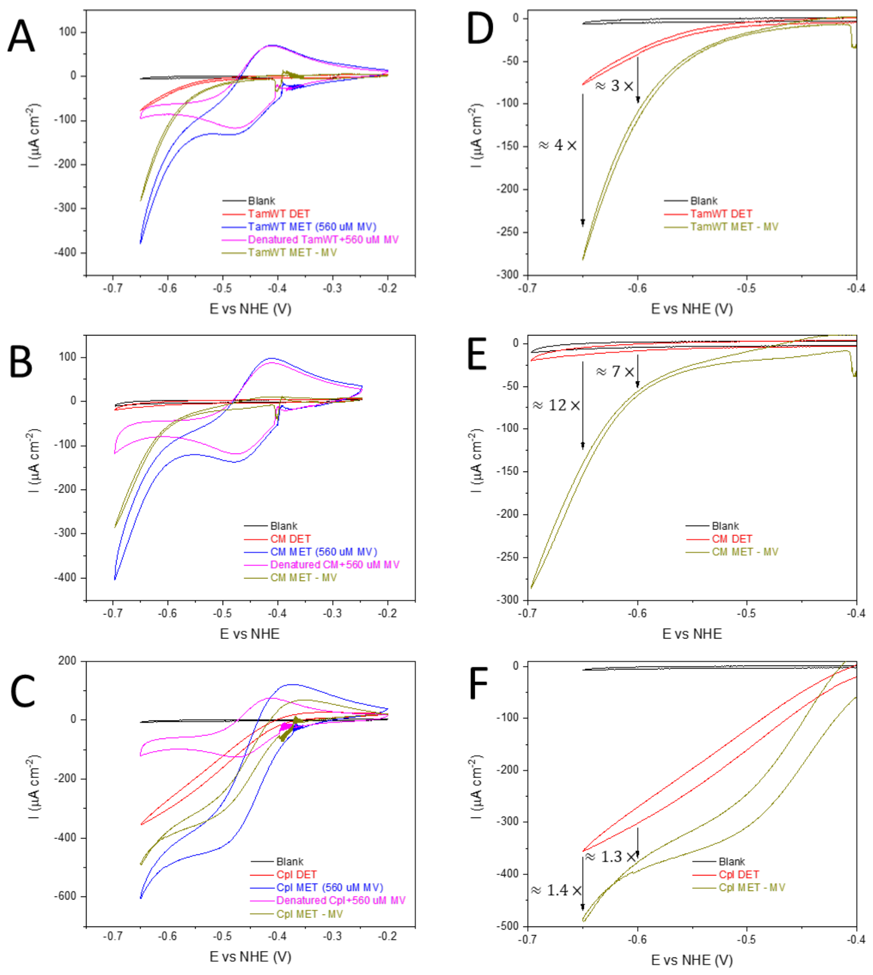

Figure S15. DET vs MET electrochemistry traces of immobilized (A) *Tam*HydS WT, (B) *Tam*HydS variant CM, and (C) *Cp*I. Experiments were performed under 1 atm of Ar, at pH 6.8, 40°C, scan rate 20 mV/s, following immobilization of the enzyme on a PGE electrode. The red line corresponds to the CV measured in the absence of a redox mediator, methyl viologen (MV), and reflects proton reduction currents by direct electron transfer (DET) from the electrode to the enzyme. The dark blue line corresponds to mediated electron transfer (MET) CVs measured in the presence of 560 µM MV.[5] The black line represents the CV subsequent to denaturation of the immobilized enzyme for 30 minutes at 90ºC, followed by buffer exchange (referred to as the background electrode or 'blank'). This 'blank' aligns closely with the CV of the bare electrode prior to enzyme immobilization, indicating minimal interference from the denaturation and buffer exchange processes on the electrochemical behavior of the electrode surface (data not shown). The pink line corresponds to the CV of denatured enzyme with the addition of 560 µM MV: $E^{0'}_{MV+/MV2+}$ = -440 mV (vs NHE), ΔE_p = 60 mV, i_{pa}/i_{pc} = 1.15 ± 0.21 (average of 4 runs). The olive line corresponds to the difference between the blue (MET) and pink (only MV) lines. Panels D−F zoom in near the region of the thermodynamic potential and the arrow label indicates the relative increase in currents from DET to MET. Two independent enzyme films were tested, with approximately 10% error for current values between films and are therefore, considered reproducible. Compared to *Cp*I, under similar conditions, the currents of *Tam*HydS **WT** and variant **CM** display more pronounced potential-dependent currents, even at high driving forces.

Figure S16. Reduction of BV^{2+} →$BV^{•+}$

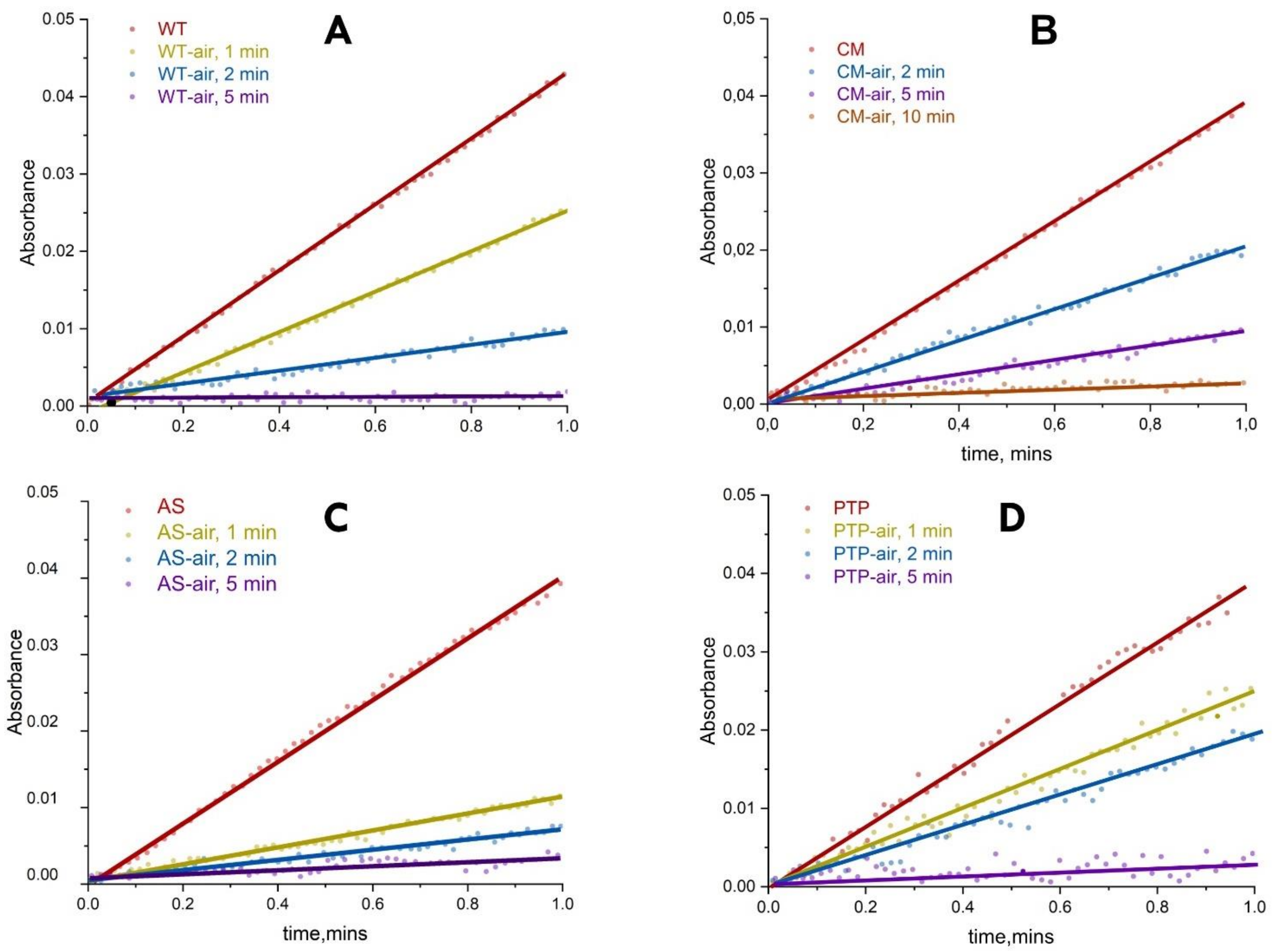

Figure S16. Reduction of BV^{2+} →$BV^{•+}$ by *Tam*HydS WT and variants before and after exposure to air at different time points. Representative UV/Vis spectra showing the increase of absorbance at 550 nm due to the reduction of benzyl viologen (ε_{550}^{red} = 9.12 mM^{-1} cm^{-1}). Panel A: **WT** (90 nM, 0.2 cm); Panel B: **CM** (90 nM, 0.2 cm); Panel C: **AS** (90 nM, 1 cm); Panel D: **PTP** (90 nM, 0.2 cm).

Figure S17. Reduction of MB^+ → LMB

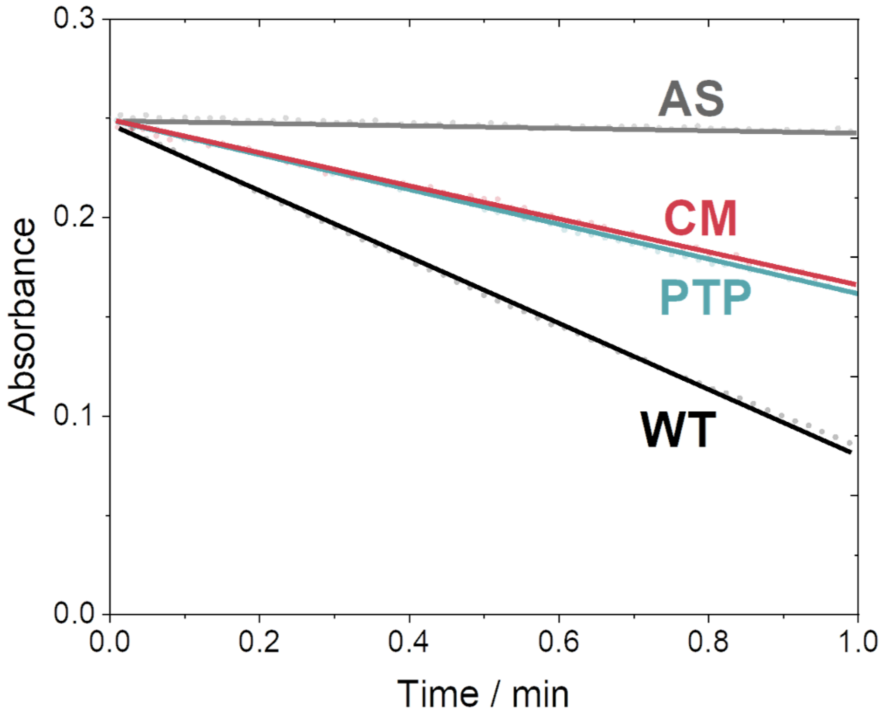

Figure S17. Reduction of Methylene Blue (MB^+) to Leucomethylene Blue (LMB) by *Tam*HydS WT and variants. Representative UV/Vis spectra showing the decrease of absorbance at 670 nm due to the reduction of methylene blue (ε_{670}^{ox} = 37.9 mM^{-1} cm^{-1}). Black: **WT** (8 nM); Red: **CM** (60 nM); Cyan: **PTP** (60 nM); Gray: **AS** (60 nM). All absorbance spectra recorded using a cuvette with a 0.2 cm path length.

Figure S18. Exponential decay curves based on *in vitro* O_2-tolerance tests

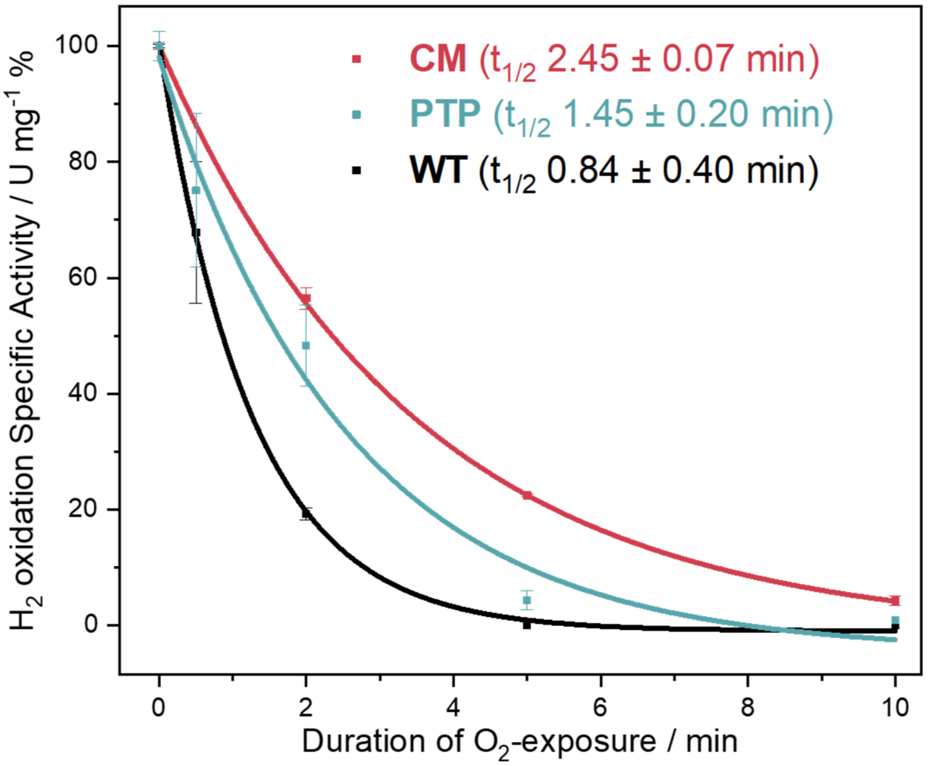

Figure S18. Exponential decay curves based on *in vitro* O_2-tolerance tests on **WT** enzyme (black) and variants **CM** (red) and **PTP** (cyan). The relative specific activities were measured after being air-exposed (approximately 21% O_2) at different time points. The maximum value (100%) is the specific activity of holo-enzyme not exposed to air. The remaining activity was assessed with the reduction of 1 mM BV in H_2-saturated 100 mM phosphate buffer, pH 6.8. Data are presented as a mean of 2-3 technical replicates (squares), with error bars showing SEM. The solid lines represent single exponential fits. The half-life values ($t_{1/2}$) in parenthesis are extracted from the best-fitted exponential decay curves.

Figure S19. Monitoring O_2 products WT vs CM vs PTP in FTIR.

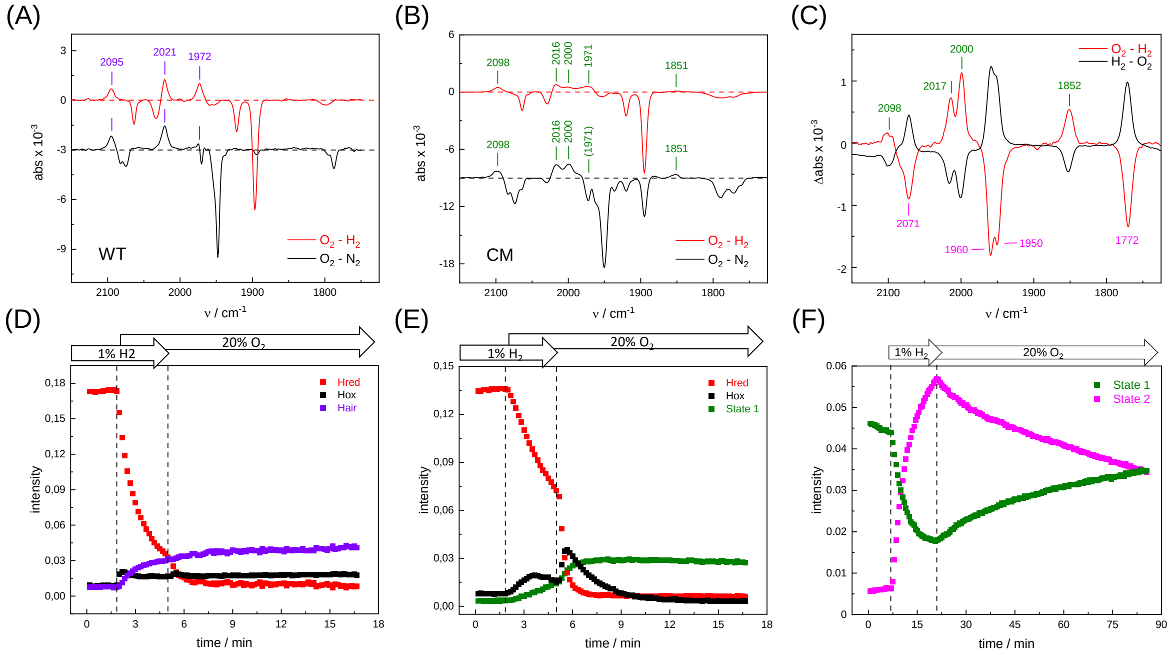

Figure S19. Reaction with 20% O_2 monitored by ATR FTIR spectroscopy. In panel **(A)–(C)**, difference spectra show the accumulation of either H_{air} (violet labels) or **State 1** (green labels), either starting from H_{ox} (black spectra) or H_{red} (red spectra). In wild-type *Tam*HydS, only H_{air} is observed **(A)** while variants **CM (B)** and **PTP (C)** exclusively form **State 1** under O_2. The traces in panel **(D)–(F)** show the reaction as a function of time and gas composition, starting with (*i*) 1% H_2 + 99% N_2, (*ii*) 1% H_2 + 79% N_2 + 20% O_2, and (*iii*) 80% N_2 + 20% O_2. Wild-type *Tam*HydS **(D)** experiences no protection against O_2 under H_2, shows only traces of H_{ox} and forms significant amounts of H_{air} (magenta traces). Variant **CM (E)** accumulates a small proportion of H_{ox}, shows minor protection against O_2 under H_2, and forms **State 1** (green traces) instead of H_{air}. Variant **PTP (F)** was reduced with 1% H_2 to form **State 2**. When reacted with 20% O_2 afterward, the difference spectrum ("O_2–H_2", red line) shows conversion of **State 2** (magenta labels) into **State 1** (green labels). When the O_2-containing atmosphere was switched back to 1% H_2, **State 2** was accumulated over **State 1** ("H_2–O_2", black line). We did not observe significant loss of cofactor integrity or an accumulation of H_{air}.

Figure S20. Calibration curves of the gas chromatograph

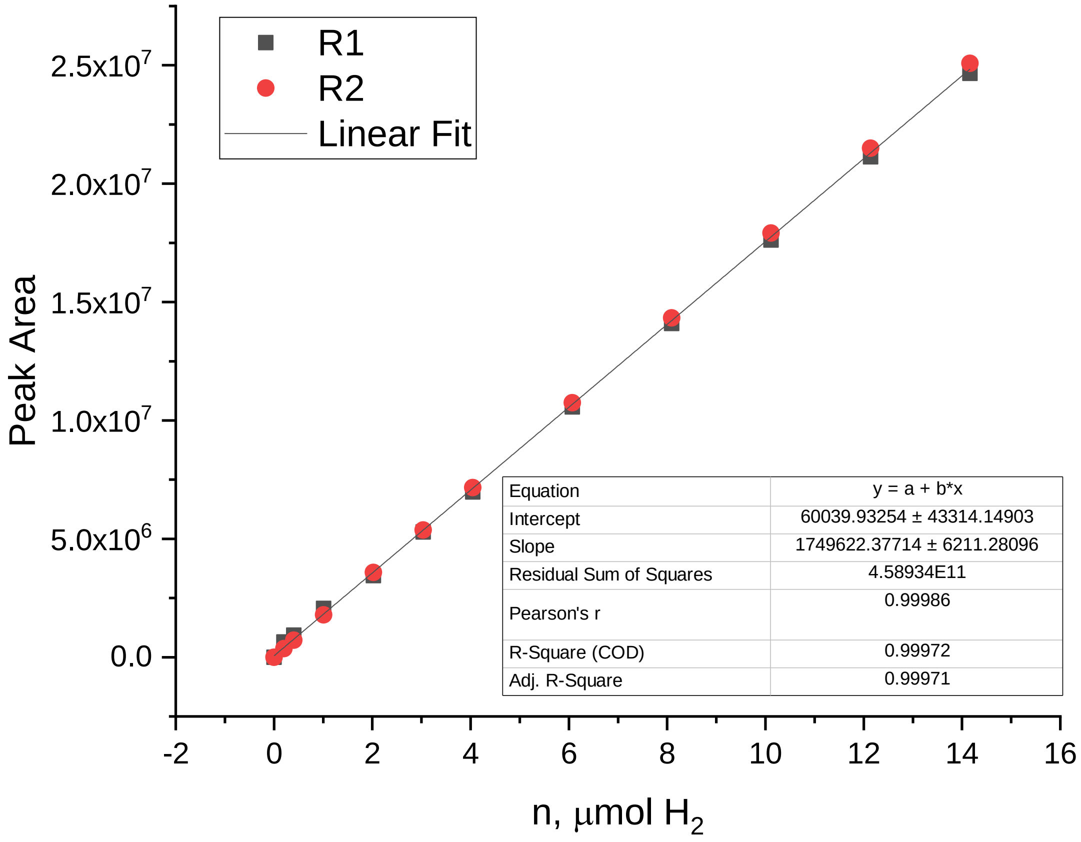

Figure S20. Calibration curves of the gas chromatograph for total H_2 produced in the headspace.

References

(1) Li, H.; Rauchfuss, T. B. Iron carbonyl sulfides, formaldehyde, and amines condense to give the proposed azadithiolate cofactor of the Fe-only hydrogenases. *J Am Chem Soc* **2002**, *124* (5), 726-727.
(2) Miyazaki, K. MEGAWHOP Cloning. In *Methods in Enzymology*, Elsevier, 2011; pp 399-406.
(3) Bradford, M. M. A rapid and sensitive method for the quantitation of microgram quantities of protein utilizing the principle of protein-dye binding. *Anal Biochem* **1976**, *72* (1-2), 248-254.
(4) Fish, W. W. Rapid colorimetric micromethod for the quantitation of complexed iron in biological samples. *Methods Enzymol* **1988**, *158*, 357-364.
(5) Gutierrez-Sanchez, C.; Olea, D.; Marques, M.; Fernandez, V. M.; Pereira, I. A.; Velez, M.; De Lacey, A. L. Oriented immobilization of a membrane-bound hydrogenase onto an electrode for direct electron transfer. *Langmuir* **2011**, *27* (10), 6449-6457.
(6) Mayhew, S. G. Potentiometric Measurement of Oxidation-Reduction Potentials. In *Flavoprotein Protocols*, Chapman, S. K., Reid, G. A. Eds.; Humana Press, 1999; pp 49-59.
(7) Fourmond, V.; Sabaty, M.; Arnoux, P.; Bertrand, P.; Pignol, D.; Leger, C. Reassessing the strategies for trapping catalytic intermediates during nitrate reductase turnover. *J Phys Chem B* **2010**, *114* (9), 3341-3347.
(8) Stripp, S. T. In Situ Infrared Spectroscopy for the Analysis of Gas-processing Metalloenzymes. *ACS Catal* **2021**, *11* (13), 7845-7862.
(9) Stoll, S. Computational Modeling and Least-Squares Fittingof EPR Spectra. In *Multifrequency Electron Paramagnetic Resonance*, 2014; pp 69-138.
(10) Stoll, S.; Schweiger, A. EasySpin, a comprehensive software package for spectral simulation and analysis in EPR. *J Magn Reson* **2006**, *178* (1), 42-55.
(11) Land, H.; Sekretareva, A.; Huang, P.; Redman, H. J.; Nemeth, B.; Polidori, N.; Meszaros, L. S.; Senger, M.; Stripp, S. T.; Berggren, G. Characterization of a putative sensory [FeFe]-hydrogenase provides new insight into the role of the active site architecture. *Chem Sci* **2020**, *11* (47), 12789-12801.
(12) Esselborn, J.; Muraki, N.; Klein, K.; Engelbrecht, V.; Metzler-Nolte, N.; Apfel, U. P.; Hofmann, E.; Kurisu, G.; Happe, T. A structural view of synthetic cofactor integration into [FeFe]-hydrogenases. *Chem Sci* **2016**, *7* (2), 959-968.
(13) Meszaros, L. S.; Ceccaldi, P.; Lorenzi, M.; Redman, H. J.; Pfitzner, E.; Heberle, J.; Senger, M.; Stripp, S. T.; Berggren, G. Spectroscopic investigations under whole-cell conditions provide new insight into the metal hydride chemistry of [FeFe]-hydrogenase. *Chem Sci* **2020**, *11* (18), 4608-4617.
(14) Kamp, C.; Silakov, A.; Winkler, M.; Reijerse, E. J.; Lubitz, W.; Happe, T. Isolation and first EPR characterization of the [FeFe]-hydrogenases from green algae. *Biochim Biophys Acta Bioenerg* **2008**, *1777* (5), 410-416.
(15) Adamska, A.; Silakov, A.; Lambertz, C.; Rudiger, O.; Happe, T.; Reijerse, E.; Lubitz, W. Identification and characterization of the "super-reduced" state of the H-cluster in [FeFe] hydrogenase: a new building block for the catalytic cycle? *Angew Chem* **2012**, *51* (46), 11458-11462.
(16) Lorent, C.; Katz, S.; Duan, J.; Kulka, C. J.; Caserta, G.; Teutloff, C.; Yadav, S.; Apfel, U. P.; Winkler, M.; Happe, T.; Horch, M.; Zebger, I. Shedding Light on Proton and Electron Dynamics in [FeFe] Hydrogenases. *J Am Chem Soc* **2020**, *142* (12), 5493-5497.
(17) Mulder, D. W.; Ratzloff, M. W.; Bruschi, M.; Greco, C.; Koonce, E.; Peters, J. W.; King, P. W. Investigations on the role of proton-coupled electron transfer in hydrogen activation by [FeFe]-hydrogenase. *J Am Chem Soc* **2014**, *136* (43), 15394-15402.
(18) Mulder, D. W.; Guo, Y.; Ratzloff, M. W.; King, P. W. Identification of a Catalytic Iron-Hydride at the H-Cluster of [FeFe]-Hydrogenase. *J Am Chem Soc* **2017**, *139* (1), 83-86.
(19) Meszaros, L. S.; Nemeth, B.; Esmieu, C.; Ceccaldi, P.; Berggren, G. *In Vivo* EPR Characterization of Semi-Synthetic [FeFe] Hydrogenases. *Angew Chem* **2018**, *57* (10), 2596-2599.
(20) Albracht, S. P.; Roseboom, W.; Hatchikian, E. C. The active site of the [FeFe]-hydrogenase from Desulfovibrio desulfuricans. I. Light sensitivity and magnetic hyperfine interactions as observed by electron paramagnetic resonance. *J Biol Inorg Chem* **2006**, *11* (1), 88-101.
(21) Bennett, B.; Lemon, B. J.; Peters, J. W. Reversible carbon monoxide binding and inhibition at the active site of the Fe-only hydrogenase. *Biochemistry* **2000**, *39* (25), 7455-7460.

(22) Chongdar, N.; Birrell, J. A.; Pawlak, K.; Sommer, C.; Reijerse, E. J.; Rudiger, O.; Lubitz, W.; Ogata, H. Unique Spectroscopic Properties of the H-Cluster in a Putative Sensory [FeFe] Hydrogenase. *J Am Chem Soc* **2018**, *140* (3), 1057-1068.
(23) Cabotaje, P. R.; Walter, K.; Zamader, A.; Huang, P.; Ho, F.; Land, H.; Senger, M.; Berggren, G. Probing Substrate Transport Effects on Enzymatic Hydrogen Catalysis: An Alternative Proton Transfer Pathway in Putatively Sensory [FeFe] Hydrogenase. *ACS Catal* **2023**, *13* (15), 10435-10446.
(24) Pandey, A. S.; Harris, T. V.; Giles, L. J.; Peters, J. W.; Szilagyi, R. K. Dithiomethylether as a ligand in the hydrogenase h-cluster. *J Am Chem Soc* **2008**, *130* (13), 4533-4540.
(25) Mukherjee, J.; Lucas, R. L.; Zart, M. K.; Powell, D. R.; Day, V. W.; Borovik, A. S. Synthesis, structure, and physical properties for a series of monomeric iron(III) hydroxo complexes with varying hydrogen-bond networks. *Inorg Chem* **2008**, *47* (13), 5780-5786.
(26) Budria, J. G.; Raugei, S.; Cavallo, L. Structure and bonding in monomeric iron(III) complexes with terminal oxo and hydroxo ligands. *Inorg Chem* **2006**, *45* (4), 1732-1738.
(27) Silakov, A.; Reijerse, E. J.; Albracht, S. P.; Hatchikian, E. C.; Lubitz, W. The electronic structure of the H-cluster in the [FeFe]-hydrogenase from Desulfovibrio desulfuricans: a Q-band 57Fe-ENDOR and HYSCORE study. *J Am Chem Soc* **2007**, *129* (37), 11447-11458.
